# Optical Properties of H-Bonded Heterotriangulene Supramolecular Polymers: Charge-Transfer Excitations Matter

**DOI:** 10.1021/acs.jpclett.4c01520

**Published:** 2024-07-25

**Authors:** Jesús Cerdá, Enrique Ortí, David Beljonne, Juan Aragó

**Affiliations:** † Laboratory for Chemistry of Novel Materials, University of Mons, Mons 7000, Belgium; ‡ Instituto de Ciencia Molecular (ICMol), Universitat de València, Catedrático José Beltrán 2, Paterna 46980, Spain

## Abstract

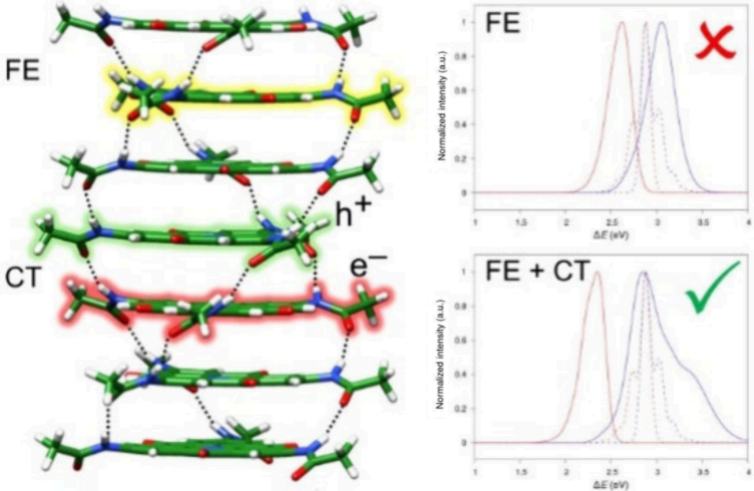

H-bonded *N*-heterotriangulene (NHT) supramolecular polymers offer a nice playground to explore the nature and dynamics of electronic excitations in low-dimensional organic nanostructures. Here, we report on a comprehensive molecular modeling of the excited-state electronic structure and optical properties of model NHT stacks, highlighting the important role of intermolecular charge-transfer (CT) excitations in shaping their optical absorption and emission lineshapes. Most importantly, we show that the coupling between the local and CT excitations, modulated by the electric fields induced by the presence of polar amide groups forming H-bonded arrays along the stacks, significantly increases the resulting hybrid exciton bandwidth. We discuss these findings in the context of the efficient transport of singlet excitons over the μm length scale reported experimentally on individual self-assembled nanofibers with molecular-scale diameter.

Supramolecular polymers (SPs) are a class of macromolecules where the monomeric building blocks are held together via noncovalent interactions (hydrogen-bonding, π–π stacking, hydrophobic interactions, etc.).^1−4^ Unlike conventional polymers, the relatively weak and reversible cohesive forces between the monomers endow SPs with a remarkable dynamic character and, consequently, they are prone to exhibit highly desirable adaptive and responsive properties (e.g., self-healing and shape memory) typical of biomolecular assemblies.^5−7^ Despite their “soft” nature, SPs sometimes form well-organized supramolecular architectures that foster the electronic and excitonic communication between the monomeric units, which is highly attractive for optoelectronic and photonic applications.^8−10^ Among these, tubular assemblies of cyanine dyes^11−14^ and supramolecular fibers based on the *N*-heterotriangulene (NHT) unit^15,16^ (Figure 1a) have been reported to exhibit singlet exciton diffusion lengths (*L*_d_) exceeding 1 μm. Similarly, highly ordered poly(di-*n*-hexylfluorene) and poly(3-hexylthiophene) (P3HT) nanofibers derived from seeded growth^17^ show much improved energy transport properties, in comparison to most organic molecular and polymeric semiconductors where the limited diffusion length in the range of ∼10 nm is a bottleneck in bulk heterojunction solar cells (requiring fine phase segregation between electron donor and acceptor cocontinuous domains).

**Figure 1:**
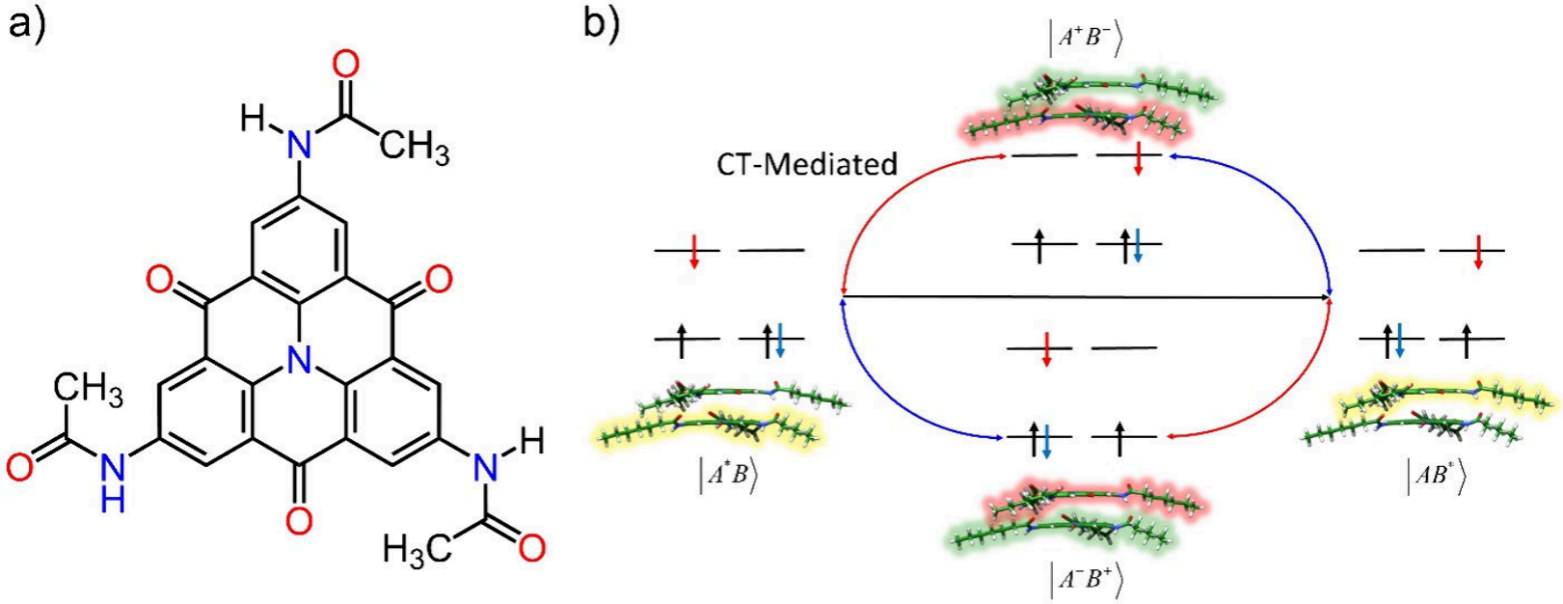
a) Chemical structure of the studied NHT monomer. b) Scheme of the CT-mediated excitonic interaction between nearest neighbor monomers.

Whereas the high diffusion coefficients and lengths measured in the P3HT nanofibers designed by the Manners group have been convincingly modeled by invoking long-range excitonic interactions and transient delocalization in dynamically disordered energy landscapes,^18,19^ there is no such a theory that explains the orders of magnitude more conductive NHT supramolecular polymers.^15,16^ Saikin and co-workers have applied robust exciton transport models, including hierarchical equations of motion (HEOM) in combination with excitonic Hamiltonians including local (Frenkel-type, FE) excitations, predicting an upper-bound to the diffusion coefficients that is at least 1 order of magnitude smaller than the experimental values.^20^ Very recently, Hildner and co-workers have theoretically characterized the exciton dynamics in NHT-based supramolecular fibers,^21^ obtaining a good agreement with the experimental exciton diffusion length. However, the authors in the proposed model (Frenkel Hamiltonian) had to assume a rather high excitonic coupling of 100 meV, not properly justified, to reproduce the experimental exciton diffusion coefficients. These interesting outcomes call for the exploration of alternative mechanisms to understand the extraordinary exciton transport exhibited in these NHT-based supramolecular aggregates.

Here, we propose that the large discrepancy between the experimental and theoretical results originates, at least partly, from the presence of low-lying intermolecular CT excitations. These mix with the FE local excitations resulting in hybrid excited states with lower exciton effective masses, hence also being more mobile. Figure 1 displays the chemical structure of the NHT monomer, as well as a sketch of the CT-mediated excitonic interactions between successive monomers along the stack.

We report below on a detailed computational investigation of the steady-state optical properties of molecularly defined NHT nanofibers, inspired by the findings from Spano and co-workers that CT excitations in close resonance with bright FE excitations can profoundly affect the shape of absorption and emission spectra in molecular aggregates.^22−24^ Our theoretical approach combines first-principles calculations with a Frenkel-CT Holstein Hamiltonian and aims at modeling the optical properties of helical NHT-based supramolecular polymers and at dissecting the nature of the resulting low-energy adiabatic states. Our findings showcase that the inclusion of CT excitations is necessary to rationalize the experimental absorption and emission features (lineshapes and lifetimes) of these supramolecular assemblies. Additionally, the calculation of the excitonic band dispersion along the nanofibers reveals a significantly reduced effective mass when CT excitations, stabilized by local electric fields induced by the H-bonded amide group network, are accounted for.

Ground-state electronic structure calculations were carried out at the density functional theory (DFT) using the B3LYP functional^25,26^ combined with the Grimme’s D3 dispersion correction and the Becke–Johnson dumping function.^27^ Excited-state calculations were performed using time-dependent DFT in its Tamm–Dancoff variant (TDA-DFT) using B3LYP and an optimally tuned (OT)^28,29^ version of the long-range corrected ωB97XD^30^ density functional (OT-ωB97XD ω = 0.15 Bohr^–1^, see Section S1 in the Supporting Information for the ω optimization). The ωB97XD functional is employed to mitigate the self-interaction error that usually leads to a significant energy underestimation of the CT states using B3LYP. All DFT and TDA-DFT calculations were performed using the 6-31G** basis set^31^ and the Gaussian16 software package (revision A03).^32^ Diabatic energies and excitonic/electronic couplings were obtained by diabatizing the outcome of the TDA-DFT calculations using the recent fragment particle-hole densities (FPHD) method developed by Zhao and co-workers,^33^ and implemented in an in-house code (a brief description of the FPHD method is given in Section S2 of the Supporting Information). To evaluate the impact that CT states have on the optical and excitonic properties of the NHT-based supramolecular aggregates, we have parametrized a Frenkel-CT Holstein Hamiltonian (see Section S3 for further details). This Hamiltonian has been successfully applied to model the Davydov splitting in oligoacene molecular crystals,^22,34^ as well as the optical properties of π–conjugated supramolecular aggregates.^35−38^

Supramolecular polymers based on *C*_3_-symmetry NHT building blocks are known to exhibit columnar and helical assemblies owing to favorable intermolecular forces between the monomeric units (π–π stacking and H-bonding).^16^ To gain more insight into the NHT-based supramolecular organization at atomistic resolution, two pentamer models were built up and, subsequently, optimized at the B3LYP-D3/6-31G** level (Figure 2). The main difference between the two models lies in the orientation of the amides. While in model I all amide groups point in the same direction along the three H-bonded amide arrays preserving the *C*_3_ symmetry, in model II one of the amide arrays is flipped and points in the opposite direction. As the local dipole moment of the amide group goes in the NH → CO direction, model II shows a smaller net dipole moment than model I due to the inverted amides in one H-bonding network. Both pentamer models hold similar structural parameters (Figure 2), with relatively short π–π contacts (∼3.25 Å) in good agreement with experimental estimates (0.33 nm),^16^ pitch rotation angles close to 35°, and NH···O bonds of *ca*. 1.81 Å, in line with analogous H-bonded helical-like supramolecular polymers.^39,40^ In terms of energetics, model II (*C*_1_ symmetry) is predicted to be slightly more stable than model I (*C*_3_ symmetry) by 1.49 kcal mol^–1^ per monomer unit.

**Figure 2:**
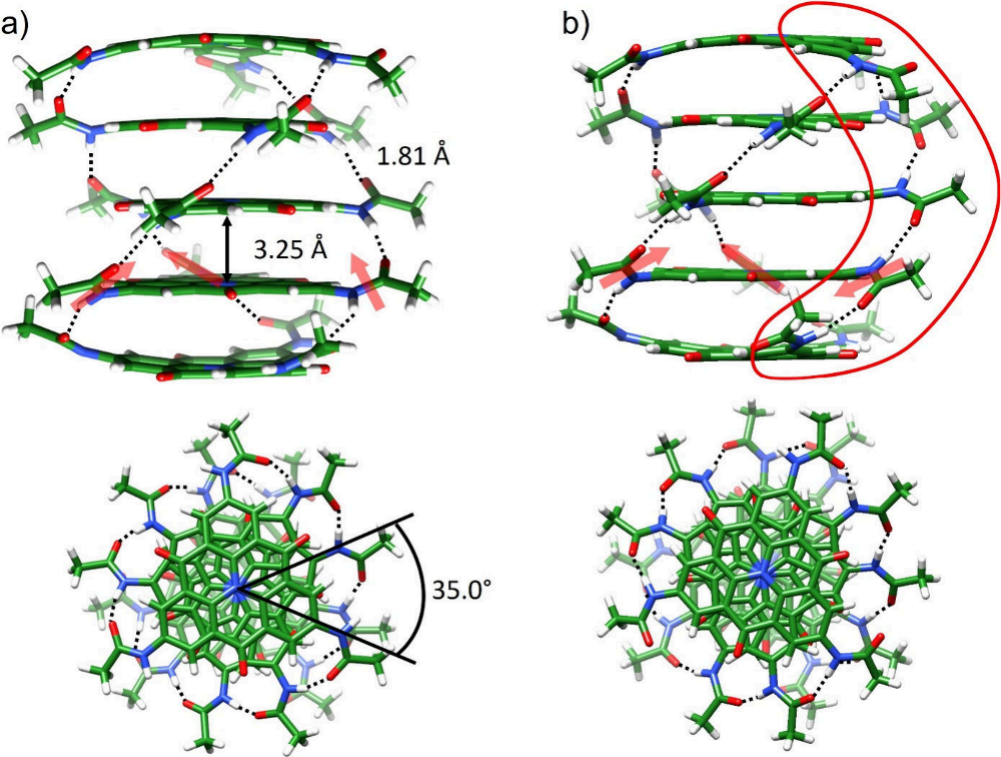
Side (top) and top (bottom) views of: a) *C*_3_-symmetric (model I) and b) nonsymmetric (model II) optimized pentamers computed at the B3LYP-D3/6-31G** level. Dotted black lines emphasize the triple H-bonded amide array. Direction of the local dipole moment of the amide groups is schematized for a molecular unit in both models. The inverted or flipped amide array is highlighted in red for model II.

Using the geometry of the central molecule and the intermolecular structural parameters inferred for the central trimer from the previously optimized pentamers, “ideal” NHT one-dimensional stacks were constructed, out of which we extracted dimers (model I and II) for electronic excited-state calculations. For sake of simplicity, *C*_3_-symmetry (model I) was only discussed to ensure localization of the molecular orbitals of each monomer due to the large dipole moment in the π-stack direction, which enables the labeling of the excited states as FE or CT (see Section S4 for details on *C*_1_-symmetry dimer). The low-lying singlet excited states for the *C*_3_-symmetry dimer were computed within the TDA-DFT approach using the B3LYP-D3 and OT-ωB97XD density functionals (see Section S4 in the Supporting Information for an extended discussion). OT-ωB97XD is preferentially used for the dimer because optimally tuned long-range density functionals are expected to provide more reasonable energy gaps between FE and CT states (Δ*E*_FE-CT_).^29,41−43^ TDA-DFT OT-ωB97XD calculations on the dimer (Table S2), hereafter denoted as AB, reveal that the lowest singlet excited states (S_1_ and S_2_) exhibit a remarkable CT character (A^–^B^+^ states), and are followed by FE-type A*B and AB* excited states (S_3_/S_4_ and S_9_/S_10_) and two CT A^+^B^–^ states (S_18_/S_19_). The effective adiabatic Δ*E*_FE-CT_ gaps with the S_1_/S_2_ states are estimated to be in the 0.4–0.7 eV window. Such a high energy difference is partly driven by the large permanent (ground-state) dipole moment characterizing model I (6.02 D), as will become clear when discussing the corresponding results for model II (2.89 D). Additionally, the two quasi-degenerate pairs of FE states show an energy splitting of ∼0.3 eV, which would correspond to a very large excitonic coupling of 150 meV in a CT-free situation. Direct calculation of the excitonic couplings using the fragment interaction scheme implemented in Gaussian16^44^ or the transition density cube approach^45^ yields much smaller values in the range 35–60 meV, already hitting toward a large effect of CT admixture.

To model the optical properties (steady-state absorption and emission spectra) of the NHT-based supramolecular polymers, a Frenkel-CT Holstein (FCTH) Hamiltonian has been used (eq S2 and Section S3 in the Supporting Information) and parametrized based on the electronic structure calculations previously performed for the different dimer models (Sections S4 and S5). When applied to the isolated monomer, our model, that accounts for the coupling of the molecular excitations to one dominant high-frequency (∼1200 cm^–1^) vibrational mode with a Huang–Rhys factor of 0.48, reproduces well the shape of the molecularly dissolved optical spectra recorded in solution (see Figure S7 and Section S6 for discussion). We next turn our attention to the NHT-based supramolecular polymers, here modeled considering 10 monomers (sites) that are sequentially rotated by an angle of 36.0° (close to that previously estimated of 35°, Figure 2), hence defining a full pitch of the helical aggregate for the decamer. This model is particularly convenient because it can be employed as a unit cell in periodic FCTH calculations. We have considered four distinct scenarios when solving the model Hamiltonian. The first and most simplified one only retains the FE excitations (thus corresponding to a simple Frenkel-Holstein model), whereas the other three include CT excitations and imply the following structural arrangements: 1) an H-bonded *C*_3_-symmetry polar configuration inspired in model I, with the three amide groups all pointing in the same direction (this configuration is also used in the calculation without CT excitations), 2) an H-bonded *C*_1_-symmetry polar configuration inspired from model II, with two out of the three amide groups aligned, and 3) a non-H-bonded apolar configuration in which the amide groups are forced to remain in the molecular plane being not able to form intermolecular H-bonds (Figure S4). The theoretically simulated absorption and emission spectra computed for these four situations are shown in Figure 3, where static disorder is incorporated via a Gaussian distribution centered on the previously computed diabatic excitation energies in a noncorrelated way with a standard deviation of 130 meV (according to the experimental work by Wittman and co-workers^16^). For comparison purposes, the absorption and emission spectra without including static disorder were also simulated (Figure S8). All the parameters used for the simulations are collected in Table S4.

**Figure 3:**
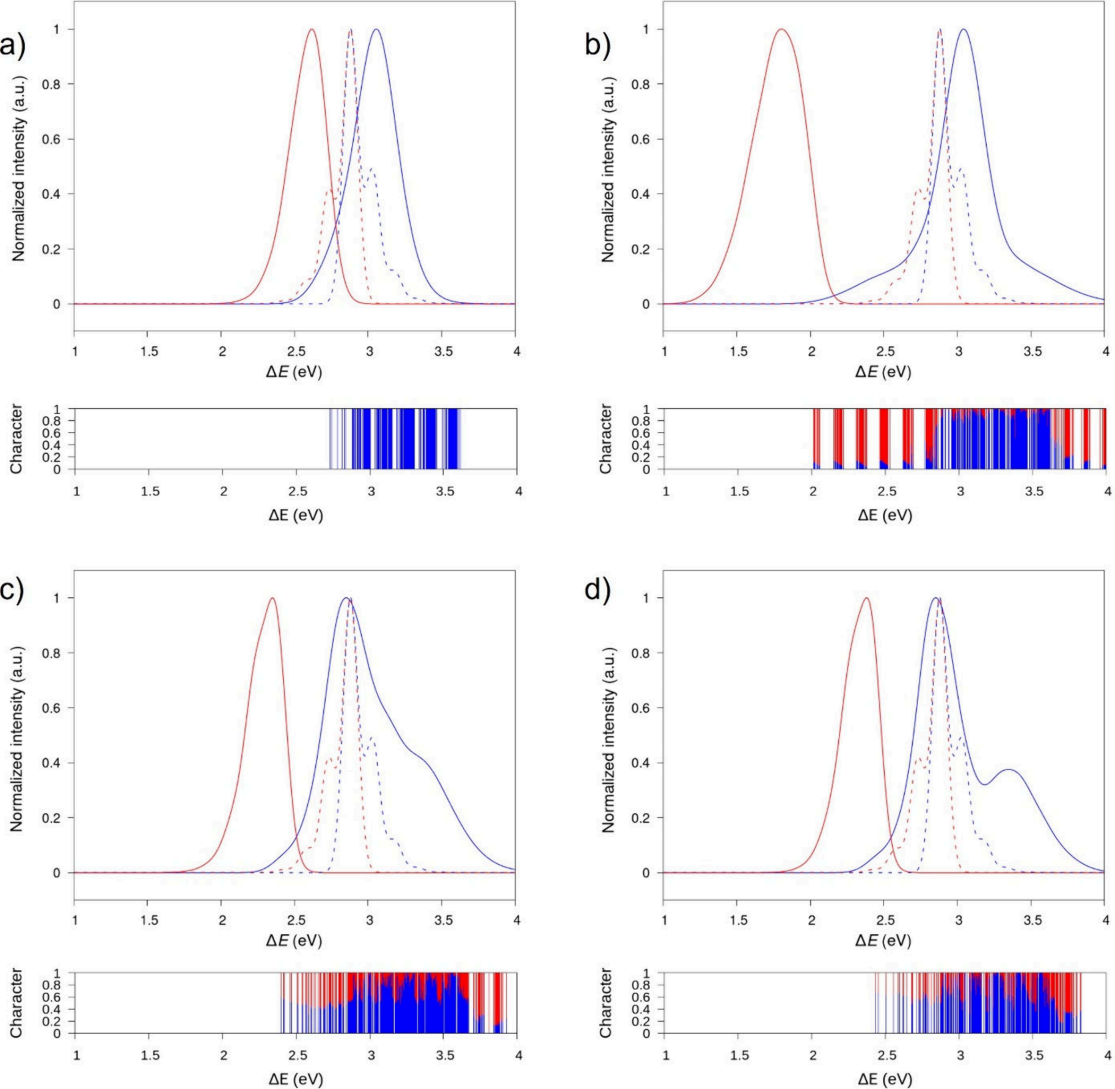
Simulated absorption (black) and emission (red) normalized spectra computed for the helical NHT-based supramolecular polymer using four different scenarios: an H-bonded *C*_3_-symmetry assembly including only FE-type excited states (a), and H-bonded *C*_3_-symmetry (b), H-bonded *C*_1_-symmetry (c), and non-H-bonded *C*_3_-symmetry apolar (d) aggregates including both FE and CT states. All spectra include static disorder. The absorption and emission spectra calculated for the monomer without static disorder (dashed lines) are included for comparison purposes. Bottom panels display the nature of the vibronic states, FE- (blue) or CT-type (red) character of the states at *k* = 0.

Figure 3 clearly illustrates that the inclusion of the CT excitations in the model Hamiltonian has a significant effect on the shape of the optical absorption and emission spectra, in line with the findings reported by Spano and co-workers in perylene bisimide supramolecular systems.^23,24,37^ For the H-bonded *C*_3_-symmetry model based purely on a localized excitations basis set (thus ignoring CT intermolecular excitations), a broad absorption (emission) band peaking at 3.05 eV (2.62 eV) is predicted with no vibrational structure (Figure 3a). In a helical supramolecular arrangement as the one investigated here, the exciton states localized on both the bottom and top of the band are optically allowed with relative intensity that depends on the actual rotation angle. The FE excitonic couplings are, however, similar to the molecular geometrical relaxation energy and the amount of static disorder, which results in the appearance of a single, broad, optical band that is blue-shifted (because of the higher oscillator strength of the exciton states at the top of the band) compared to the monomer spectrum (by *ca*. 0.18 eV). The steady-state emission is also featureless and Stokes-shifted from the optical absorption maximum because of inhomogeneous broadening. Additionally, the thermally averaged radiative lifetime is estimated to have a value of 25 ns (see Section S3 for details), which is more than 1 order of magnitude smaller than the 419 ns obtained from the measured time-dependent photoluminescence spectra and quantum yields.^16^

When including CT excitations in the Hamiltonian of the H-bonded *C*_3_-symmetry polar arrangement, the absorption spectrum presents a maximum at almost the same energy position (3.04 eV) as in the CT-free model, but now features long tails at both the low- and high-energy regions. These tails arise from the weak admixture of the optically active FE excitations with the CT-like excitations (with the diabatic CT states bracketing the diabatic FE states in the energy scale). The emission band is broader and largely red-shifted (with maximum intensity at *ca*. 1.80 eV) compared to the pure FE model, with a Stokes shift now reaching 1.25 eV. The low-energy emission in fact originates from vibronic states that hold a dominant CT character and, therefore, also features an extremely long radiative lifetime of 2844 ns, now much larger than the experimental value in the aggregate. In contrast, the H-bonded *C*_1_-symmetry polar and the purely π-stacked *C*_3_-symmetry apolar aggregates with smaller diabatic Δ*E*_FE-CT_ gaps (Table S4) result in a narrower spectrum of adiabatic excited states of intimately mixed FE-CT character (Figure 3c-d). Interestingly, the spectral shift in the absorption maximum going from the isolated monomer to the aggregate is now small (and slightly to the red), because of close cancellation between blue-shifting FE excitonic interactions and red-shifting FE-CT mixing. Light emission from the thermalized exciton band bottom is characterized by a radiative lifetime of 209 ns for H-bonded *C*_1_-symmetry polar aggregate (139 ns for purely π-stacked *C*_3_-symmetry apolar aggregate), in very good agreement with experiment. The calculated Stokes shift of ∼0.48 eV also nicely matches the measured value (0.48 eV, Figure 4).

**Figure 4:**
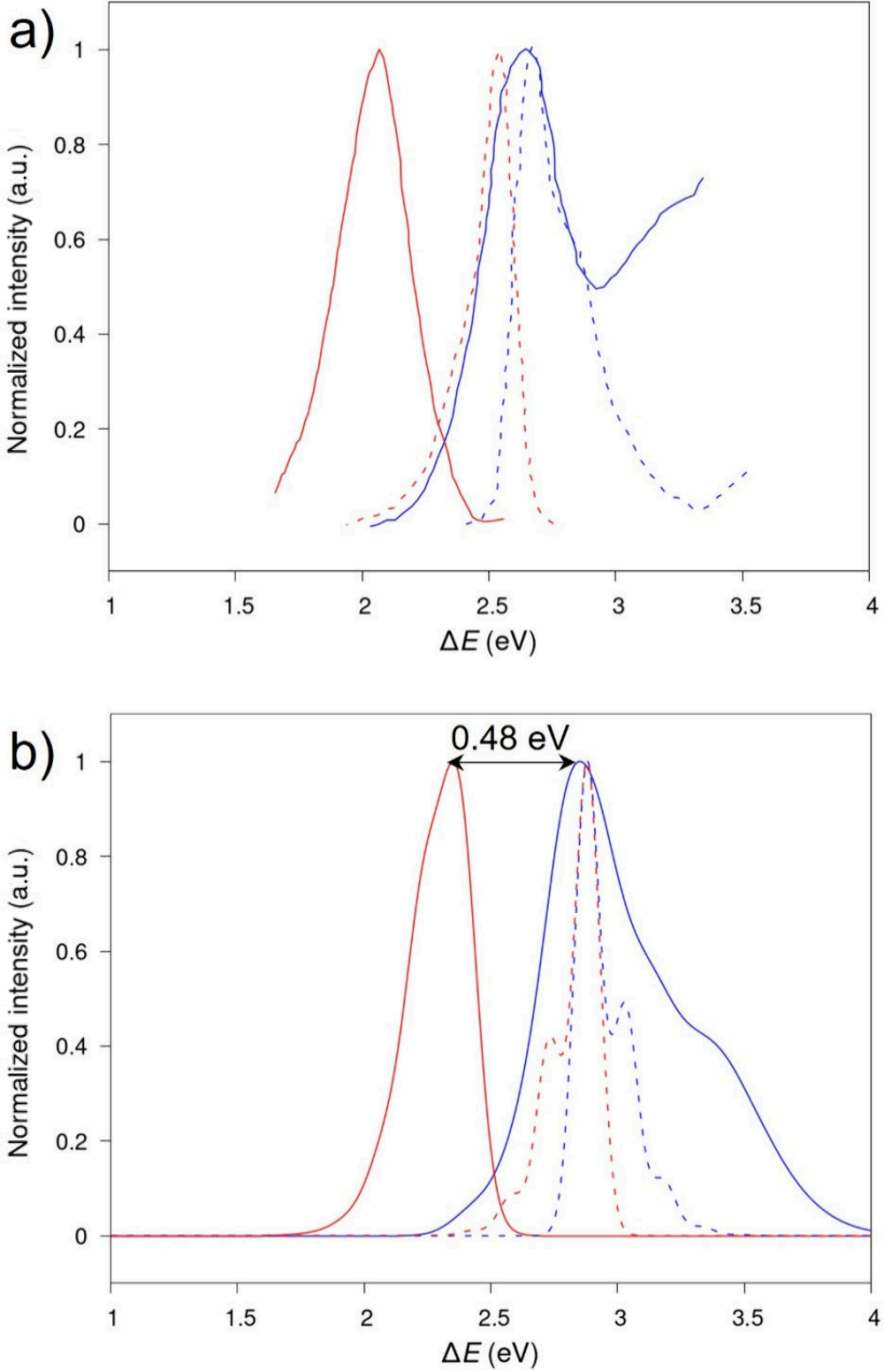
a) Normalized absorption (blue) and emission (red) spectra experimentally registered for a helical NHT-based supramolecular polymer. b) Normalized absorption (blue) and emission (red) spectra computed for the H-bonded *C*_1_-symmetry assembly. Dashed lines correspond to the spectra of the dissolved monomer. Experimental spectra were digitalized from ref (16).

The results shown in Figure 3 for the different models used to simulate the NHT supramolecular polymer should be compared with the optical spectra experimentally recorded for molecular-scale nanofibers displayed in Figure 4a. The two variants of the helical H-bonded *C*_3_-symmetry assembly (with only FE-type excited states and including all excitations) yield simulated absorption spectra (Figure 3a-b) in poor agreement with experiment. First, these models predict the absorption maximum in the aggregate to be blue-shifted by ∼0.18 eV compared to the monomer spectrum. This contrasts with the experimental results showing a corresponding slight red shift (∼0.1 eV). Second, the Stokes shift of 1.25 eV calculated by the model including the CT excitations (Figure 3b) is largely overestimated with respect to the experimental value of 0.57 eV. Conversely, the use of the FCTH model for either the polar H-bonded *C*_1_-symmetry aggregate and the apolar non-H-bonded *C*_3_-symmetry aggregate (Figure 3c-d) yields spectral properties in line with the experimental findings. Namely: (i) the absorption spectrum maximum is slightly red-shifted upon aggregation (*ca*. 0.05 eV) and (ii) the emission is Stokes shifted by ∼0.48 eV. Of course, one should keep in mind that the formation of an H-bonded array between the amide groups is what stabilizes π-stacked NHT arrangements in specific solvents, hence out of the two models of Figure 3c-d that provide results in good agreement with experiment, the polar H-bonded *C*_1_-symmetry self-assembly is also the most thermodynamically stable and therefore the most realistic.^16^ We would like to conclude this section by stressing the fact that our FCTH Hamiltonian, when applied to the helical H-bonded *C*_1_-symmetry supramolecular aggregate, nicely reproduces both the overall lineshapes of the absorption and emission spectra as well as the main spectral changes experimentally observed when going from the isolated molecule to the nanofibers and reveals the importance of including the admixture between FE and CT excitations to describe the nature of the relevant adiabatic singlet excited states.

As a first assessment of the impact of CT excitations on energy transport in helical NHT-based supramolecular polymers, we have computed the exciton band structure for the previous four electronic situations mimicking the plausible aggregate models. The same Hamiltonian (eq S2) as that adopted for the simulation of the optical properties was used to calculate the band structure, but now a single molecular site was employed as the unit cell. The small unit cell takes advantage of the high degree of periodicity in electronic interactions compared to transition dipole moments, thereby avoiding band folding in large supercells and simplifying the analysis.

Figure 5 depicts the pure electronic band diagrams along with the band character at each *k*-point for the four supramolecular polymer models. For the helical H-bonded *C*_3_-symmetry assembly (reference model) with only FE-type excited states (Figure 5a), the band energy dispersion (*E*_d_) is found to be 0.21 eV, which in a simple 1D tight-binding model would correspond to four times the excitonic coupling. The effective mass (*m*_eff_), computed at the bottom of the lowest energy band as *m*_eff_ = *ℏ*^2^ /(∂^2^*E*/∂*k*^2^), where *k* is the reciprocal space coordinate, is estimated to be 7.0 *m*_e_. When CT states are included, the picture changes significantly. For the H-bonded *C*_3_-symmetry polar assembly (Figure 5b), the lowest-energy excitonic band has a fairly limited FE character (∼20%), irrespective of the *k*-point sampling, owing to the substantial diabatic Δ*E*_FE-CT_ energy gap. As a result, this model predicts a decrease in *E*_d_ (0.12 eV) and an accompanying raise in *m*_eff_ up to 10.3 *m*_e_. Thus, the bottom states in the manifold spectrum are heavy carriers and are expected to act as CT-like trapping states. Meanwhile, for the H-bonded *C*_1_-symmetry polar aggregate (Figure 5c) and the non-H-bonded *C*_3_-symmetry apolar aggregate (Figure 5d), the lowest-energy band evolves from a mainly FE-type character (*ca*. 60%) at the limit of the Brillouin zone, from where emission takes place, to a largely contributed CT-type nature at the center of the Brillouin zone (*k* = 0). The effective FE-CT mixing results in an increase in *E*_d_ values to 0.29 and 0.33 eV and a lower effective exciton mass of 5.3 and 5.0 *m*_e_, respectively. We thus anticipate that the formation of hybrid FE-CT states at the bottom of the exciton band in the H-bonded *C*_1_-symmetry polar model, which was previously validated as being the most realistic, favors faster exciton diffusion along the NHT helical stacks.

**Figure 5:**
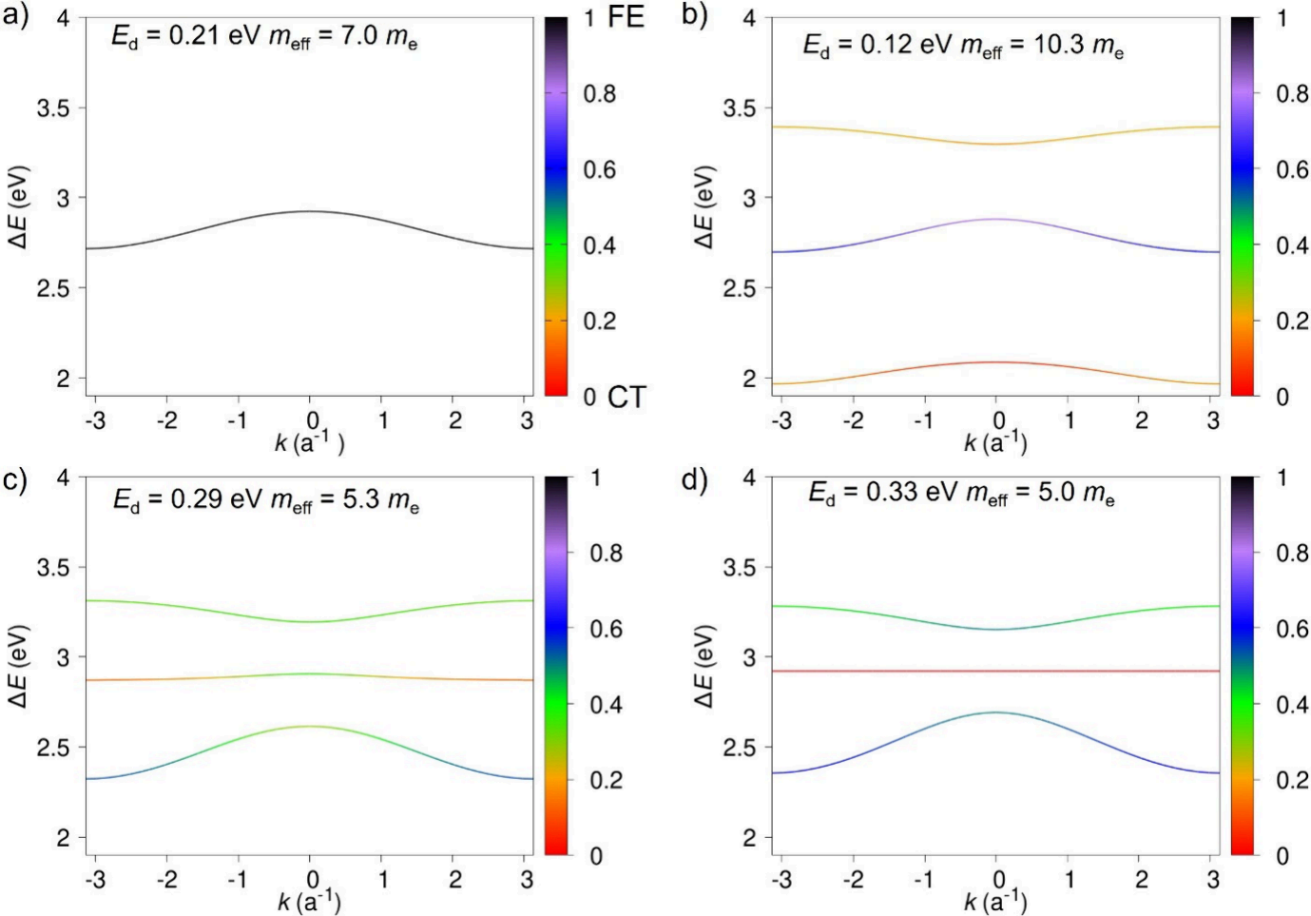
Band diagram of the electronic states calculated for the helical NHT aggregate using different models: a) H-bonded *C*_3_-symmetry polar without including CT states, b) H-bonded *C*_3_-symmetry polar, c) H-bonded *C*_1_-symmetry polar, and d) non-H-bonded *C*_3_-symmetry apolar, with the last three including CT states. Color scale represents the degree of FE character of the band at each *k*-point.

In a final step, we have extended the calculations to account for vibrational dressing of the mixed electronic states (Figure S9). Upon inclusion of vibrations, there are no remarkable changes regarding the nature of the lowest-energy excitonic band, which shows the same CT-like character for the helical H-bonded *C*_3_-symmetry polar case, while a high FE-type character is maintained throughout the lowest-energy vibration-excitonic band for the H-bonded *C*_1_-symmetry polar and the non-H-bonded *C*_3_-symmetry apolar aggregate models. Although *E*_d_ decreases compared to the pure electronic picture (this is expected since the coupling matrix elements responsible for band dispersion are weighted by Franck–Condon integrals), a similar trend is obtained; i.e., the appearance of a significant FE-CT mixing due to a small diabatic Δ*E*_FE-CT_ energy gap determines an increase (decrease) in *E*_d_ (*m*_eff_).

From the vibronic band structure, we have computed the density of states (DOS) for the four supramolecular polymer models (Figure S10). For the four models, we predict the maximum DOS approximately at 3.5 eV, much higher energies than those obtained for all the maximum peaks in the absorption spectra of the supramolecular polymer models (Figure 3). This comes from the large number of the two-particle FE-type basis functions, which are optically dark and are found at the same energy for the four models. Note that the number of two-particle functions grows up in energy owing to the increasing of vibrational quanta. Turning our attention to the low-energy part of DOS, we show that some peaks appears when CT states are added to the models, especially when these CT states are placed at small energies (e.g., H-bonded *C*_3_-symmetry aggregate). These hybrid low-energy states with a marked CT character are mainly responsible for the spectra shape and red-shifted emission compared with the model without CT states (Figures 3 and S10).

To sum up, we have evaluated the impact of intermolecular CT excitations on the optical properties of an *N*-heterotriangulene supramolecular fiber based on an amide H-bonded network. The energy mismatch between the local (molecular) and CT excitations is highly sensitive to the relative orientation of the amide groups along the 1D columns. In the most realistic model, where 2 out of the 3 amide groups per monomer are aligned, the simulated optical absorption and emission spectra are in remarkable agreement with the experimental data, both in terms of spectral shapes and the magnitude of the Stokes shift. Calculation of the exciton band structure reveals that partial admixture of CT excitations into the wave function of the bottom band states reduces their effective masses, likely leading to higher exciton diffusion coefficients. While a definitive conclusion would require running nonadiabatic molecular dynamics simulations accounting on an equal footing for the excitonic interactions, the exciton–phonon couplings and the energetic disorder (now in progress), we propose that the existence of relatively light Frenkel-CT hybrid states (small effective masses) in these supramolecular polymers is one of the key ingredients at the origin of the abnormally high exciton diffusion length measured in NHT-based supramolecular single fibers.

## Supporting Information Available

Brief description of the long-range optimization functional procedure; brief description of the diabatization method; methodology used to compute optical properties and excitonic band structure of aggregates; details of the excited states computed for the monomer and dimer building blocks in the adiabatic and diabatic picture as well as supplementary figures (PDF)
Transparent Peer Review report available (PDF)
