# Supplementary material for: Optical Properties of H-Bonded Heterotriangulene Supramolecular Polymers: Charge-Transfer Excitations Matter
Source: J Phys Chem Lett. 2024 Jul 25;15(30):7814–21. doi: 10.1021/acs.jpclett.4c01520 (PMC11299171; doi:10.1021/acs.jpclett.4c01520)
Supplement: Supplementary file 1 — jz4c01520_si_001.pdf [file jz4c01520_si_001.pdf]

Supporting Information

Optical Properties of H-bonded Heterotriangulene Supramolecular Polymers: Charge-Transfer Excitations Matter

Jesús Cerdá[a], Enrique Ortí[b], David Beljonne[a]*, Juan Aragó[b]*

[a]*Laboratory for Chemistry of Novel Materials, University of Mons, Mons 7000, Belgium.*

Email: David.Beljonne@umons.ac.be

[b]*Instituto de ciencia molecular (ICMol), Universidad de Valencia, Paterna, 46980, Spain.*

Email: juan.arago@uv.es

Contents

S1 Tunning of the long-range corrected density functional..................................3
S2 Diabatization based on the fragment particle–hole densities (FPHD) 4
S3 Optical and electronic properties of aggregates 5
S4 Electronic structure of monomer and dimer systems 8
S5 Diabatic model Hamiltonian...16
S6 Simulation of absorption and emission spectra of NHT-based systems........19
S7 Band structure and exciton transport properties for the model aggregates.22
S8 References 24

S1 Tunning of the long-range corrected density functional

Long-range corrected (LC) functionals split the exchange term in a short-range and a long-range component. The first component is represented by the exchange expression of the own density functional whereas the second is evaluated with an "exact" Hartree-Fock exchange expression.[1] LC functionals significantly depend on the ω parameter, which measures the threshold distance for the short-range and long-range region. Optimization of the ω parameter for an LC functional applied to a donor−acceptor (D−A) supramolecular complex is highly recommended to obtain acceptable energies for the CT excited states and satisfy the Mulliken rule $E_{CT} = IP + EA - 1/r$,[1] where IP, EA, and r correspond to the ionization potential, the electron affinity, and the distance between the donor and acceptor units, respectively. Here, we have performed an optimization of ω by minimizing the $J(\omega)$ function (Eq. S1) that provides the deviation of the HOMO energy ($\varepsilon_{HOMO}^{\omega,S}$) of the neutral system with respect to the IP plus the deviation of the HOMO energy of the anion system with respect to the EA.

$$J(\omega) = \left|\varepsilon_{HOMO}^{\omega,S} + IP\right| + \left|\varepsilon_{HOMO}^{\omega,S^-} + EA\right| \qquad \text{S1}$$

Figure S1 provides the tuning of the ω parameter for the NHT molecule using the LC ωB97XD functional[2] and the 6-31G** basis set.[3]

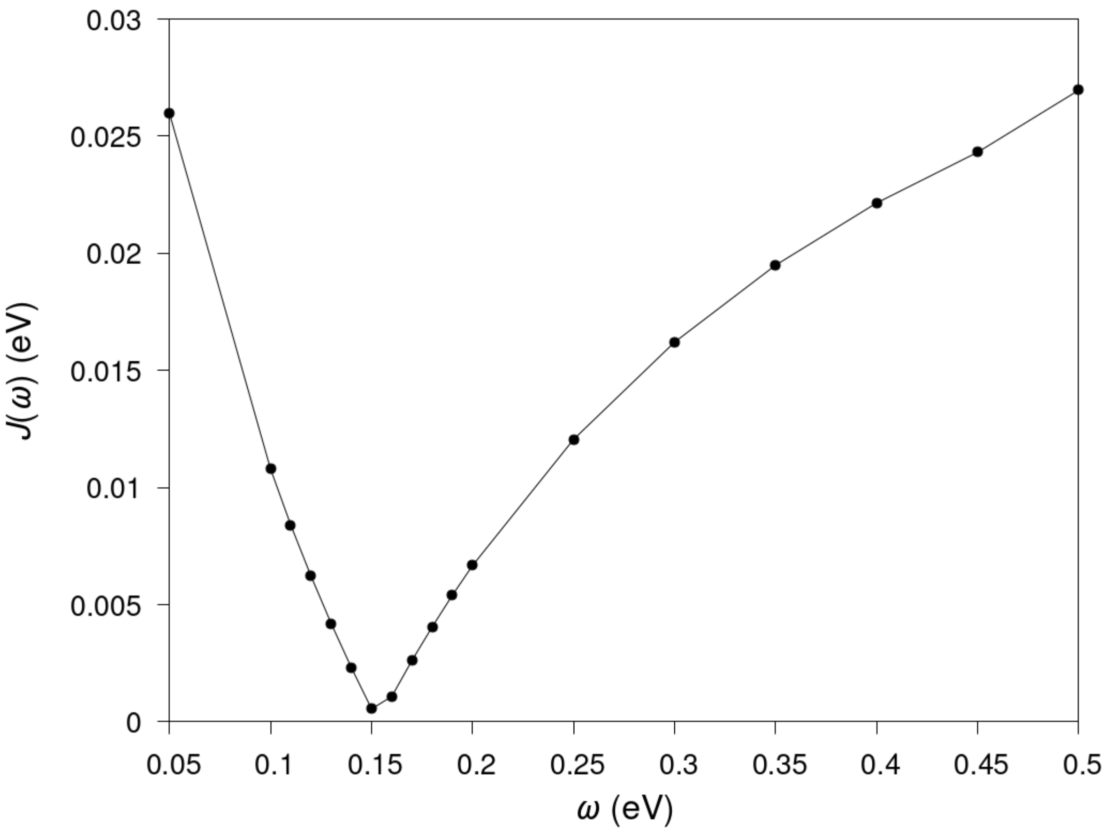

Figure S1. Plot of the $J(\omega)$ function for the NHT molecule using the ωB97XD functional and the 6-31G** basis set.

S2 Diabatization based on the fragment particle–hole densities (FPHD)

The diabatization scheme[4] based on the fragment particle–hole densities is a method developed to include simultaneously local excited states (Frenkel states, FE) and charge-transfer (CT) states, which is a non-trivial task. For instance, in the simple case of a dimer, four type of diabatic states can be found: two FE states and two CT-states. By using the hole and electron population of each fragment, the FPHD diabatization allows to set a property that univocally describes both the local and the CT states. That means that $2N$ matrices (holes and electrons) are simultaneously used, where N is the number of fragments. Figure S2 displays the 4 diabatic property matrices associated to the diabatic states. As in the adiabatic representation the four matrices collecting the hole and electron transition density populations are non-diagonal, the FPHD method requires of a unique unitary transformation matrix (**C**) that performs the adiabatic-to-diabatic transformation to the $2N$ property matrices simultaneously.

The diabatic states are those that have a maximum localization of the hole and electron populations. This maximum localization is achieved through simultaneous diagonalization of the $2N$ matrices, what is accomplished by the Jacobi sweep algorithm.[5] Once the adiabatic matrices are diagonalized, the diabatic states can be classified according to their nature, and each subspace of a specific nature must be diagonalized to ensure that the couplings between states of the same nature are zero. This procedure thus implies the diabatization between local FE states or CT states of equivalent nature, and therefore provides simultaneously electronic and excitonic couplings.

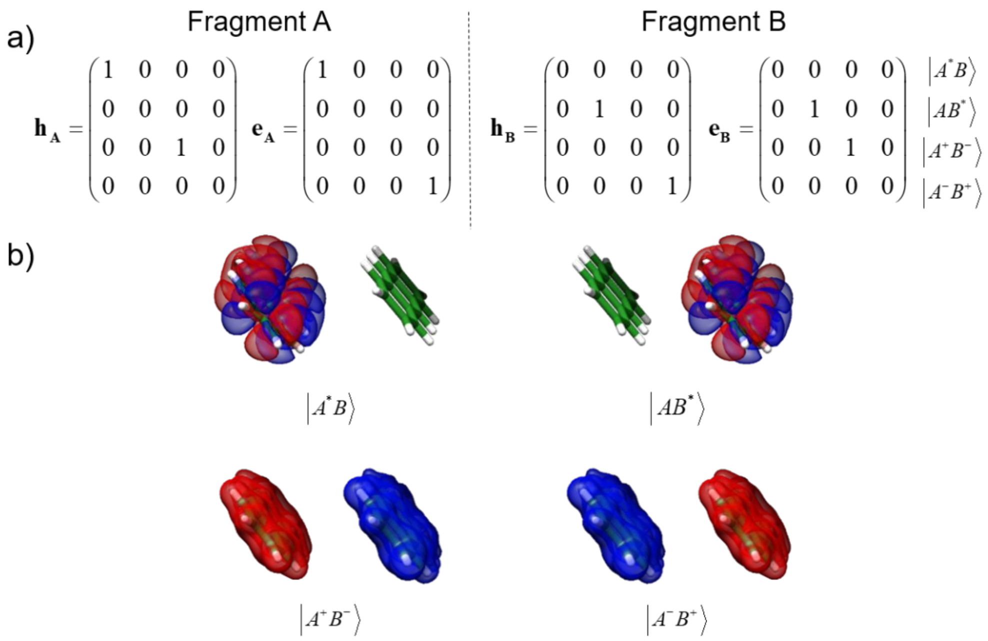

Figure S2. a) Diabatic matrix representation of the hole (**h**) and electron (**e**) transition density populations of each fragment (A or B) for the four diabatic states. Labels indicating the diabatic states are provided at the right side. b) Hole (red) and particle (blue) transition densities are represented for each state, using an anthracene dimer as example model.

S3 Optical and electronic properties of aggregates

The Frenkel-CT Holstein (FCTH) Hamiltonian used to simulate the optical and electronic properties of the NHT-based molecular aggregates can be written as:

$$\hat{H} = \hat{H}_{FE} + \hat{H}_{CT} + \hat{H}_{FE-CT} + \hat{H}_{Vib} \qquad \text{S2}$$

Where the Frenkel part in the nearest neighbor's approximation reads as:

$$\hat{H}_{FE} = \sum_{n=1}^{N} \left[E + \omega_0 \left(\lambda_* \left(b_n^\dagger + b_n \right) + \lambda_*^2 \right) \right] |n\rangle\langle n| + \sum_{n=1}^{N-1} J |n+1\rangle\langle n| + h.c., \qquad \text{S3}$$

where E represents the energy of a Frenkel exciton in the site n in the aggregate, J is the excitonic coupling, ω_0 the frequency of the effective normal mode that modulates the energy of the excited site and λ_*^2 is the Huang-Rhys factor of the Frenkel state (S_1) that measures the relative shift with respect to the S_0. $b_n^\dagger$ and b_n are the creation and annihilation operators of a vibrational quantum in the unshifted S_0 nuclear potential well on-site n.

Similarly, we define a CT Hamiltonian (Eq. S4), which is diagonal since we assume only interaction between consecutive sites, as:

$$\hat{H}_{CT} = \sum_{n=1}^{N-1} \left[\begin{array}{l} \left(E_{+-} + \omega_0 \left(\lambda_+ \left(b_n^\dagger + b_n \right) + \lambda_- \left(b_{n+1}^\dagger + b_{n+1} \right) + \lambda_+^2 + \lambda_-^2 \right) |n^+, n+1^-\rangle\langle n^+, n+1^-| \right) + \\ \left(E_{-+} + \omega_0 \left(\lambda_+ \left(b_{n+1}^\dagger + b_{n+1} \right) + \lambda_- \left(b_n^\dagger + b_n \right) + \lambda_+^2 + \lambda_-^2 \right) |n^-, n+1^+\rangle\langle n^-, n+1^+| \right) \end{array} \right]. \qquad \text{S4}$$

E_{+-} and E_{-+} are the energy of the $|A^+B^-\rangle$ and $|A^-B^+\rangle$ CT states, and λ_+^2 and λ_-^2 are the Huang-Rhys factors associated to the cation and anion, respectively.

Eq. S5 contains the coupling elements between the FE and CT states, where t_h and t_e are defined as the Hamiltonian coupling elements between FE and CT states with a hole or electron of difference, respectively.

$$\hat{H}_{FE-CT} = \sum_{n=1}^{N-1} \left[t_\mathrm{e} |n\rangle\langle n^+, n+1^-| + t_\mathrm{h} |n\rangle\langle n^-, n+1^+| \right] + h.c. \qquad \text{S5}$$

Lastly, the last term of the Hamiltonian just contains the energy of the vibrational quanta in the ground state potential energy surface (Eq. S6):

$$\hat{H}_{Vib} = \omega_0 \sum_{n=1}^{N} b_n^\dagger b_n \, . \qquad \text{S6}$$

The full Hamiltonian is expressed in a delocalized basis set including one- and two-particle states for FE excitons and two-particle states for CT states. Therefore, the ath eigenstate wave vector is expanded as:

$$\left|\Psi_{a,k}\right\rangle = e^{ikn}\sum_{n,v}\left[\begin{array}{l} c_{n,v_n}^{a,k}\left|n,v_n\right\rangle + \sum_{m\neq n,w}\left(c_{n,v_n,m,w_m}^{a,k}\left|n,v_n,m,w_m\right\rangle\right) + \\ \sum_{w}\left(c_{n^+,v_n,n+1^-,w_{n+1}}^{a,k}\left|n^+,v_n,n+1^-,w_{n+1}\right\rangle + c_{n^-,v_n,n+1^+,w_{n+1}}^{a,k}\left|n^-,v_n,n+1^+,w_{n+1}\right\rangle\right)\end{array}\right]. \quad \text{S7}$$

$\left|n,v_n\right\rangle$ and $\left|n,v_n,m,v_m\right\rangle$ are the local one- and two-particle FE states, where the exciton is at site *n*, and v_n and w_m are the vibrational quanta in sites *n* and *m*, respectively, and $\left|n^+,v_n,n+1^-,w_{n+1}\right\rangle$ and $\left|n^-,v_n,n+1^+,w_{n+1}\right\rangle$ are the corresponding two-particle CT states. In this contribution, we use a basis cutoff with the maximum number of quanta $\left(v_n + w_m\right)$ is limited to 5. The intensity of the absorption spectra is computed as:

$$I_{abs}\left(\omega\right) = \sum_{a,k=0}\left|\mu_{0a,k=0}\right|^2 e^{-\frac{\left(\omega-\omega_{a,k=0}\right)^2}{2\sigma^2}}, \quad \text{S8}$$

where $\mu_{0a,k=0}$ is the transition dipole moment between the vibrational ground state and eigenstate *a* at the high symmetry *k* point, and the exponential part adds a homogeneous broadening with a standard deviation (σ) of 0.05 eV. The transition dipole moments are estimated by rotating the diabatic transition dipole moments with the eigenvector matrix from diagonalizing the model Hamiltonian from Eqs. S2-S6. In a similar way, the emission intensity is computed as:

$$I_{emi}\left(\omega\right) = \sum_{a,k=0} Q\left(a\right)\sum_{w}\left|\mu_{wa,k=0}\right|^2\left(1 - v\omega_0/\omega_{a,k=0}\right)^3 e^{-\frac{\left(\omega-\omega_{a,k=0}-v\omega_{0,k=0}\right)^2}{2\sigma^2}}, \quad \text{S9}$$

$$Q\left(a\right) = e^{-\left(\omega_{a,k=0}-\omega_{a=0,k=0}\right)/kT} / \sum_{a} e^{-\left(\omega_{a,k=0}-\omega_{a=0,k=0}\right)/kT}, \quad \text{S10}$$

where the summation over all the receiving states, denoted with symbol w, is incorporated. The receiving states are the one- and two-particle vibronic basis of the electronic ground state. Thus, $\mu_{wa,k=0}$ is the transition dipole moment from eigenstate *a* to the w vibronic ground state with a total number of v vibrational quanta. The emission intensities are thermalized by weighting the intensities according to a Boltzmann distribution (Eq. S10) on the emissive states.

To include static disorder in the simulation of the optical properties, we compute the absorption and emission spectra of 1000 replicas where the values of E, E_{+-}, and E_{-+} are

randomized independently according to a Gaussian distribution with the mean centered in the reference values (see Table S4 below) and a standard deviation of 130 meV in a non-correlated manner. Therefore, all local (E) and CT (E_{+-} and E_{-+}) excitations are likely to be different at each realization and in between realizations. Finally, we obtain the average spectra of the 1000 realizations.

Radiative lifetimes (τ) are estimated as an average of the lifetime computed for each realization ($\tau_i = 1/k_{rad,i}$, Eq. S11), and the radiative decay rate $k_{rad,i}$ is computed as a summation over the thermally weighted emission line spectrum (Eq. S12).

$$\langle \tau \rangle = \frac{1}{N}\sum_{i=1}^{N}\frac{1}{k_{rad,i}} \qquad \text{S11}$$

$$k_{rad,i} = \frac{\sum_{a,k=0} Q(a)\sum_{w}\left|\mu_{wa,k=0}\right|^2\left(1-v\omega_0/\omega_{a,k=0}\right)^3\omega_{a,k=0}^3}{3\varepsilon_0\pi\hbar^4c^3} \qquad \text{S12}$$

In Eq. S12, $\omega_{a,k=0}$ is the energy of the emissive state, ε_0 the vacuum dielectric permittivity, $\hbar$ the reduced Planck constant, and c the speed of light. Note that Eq. S12 requires that $\omega_{a,k=0}$ and ω_0 should be in the same energy units.

S4 Electronic structure of monomer and dimer systems

The electronic structure and the excited states of a N-centered heterotriangulene (NHT) symmetrically substituted with three acetamide groups (Figure S3a) were studied at the B3LYP-D3/6–31G** level after its previous geometry optimization within C_3 symmetry restrictions. Figure S3b displays the computed frontier molecular orbitals leading to a single HOMO energy level and a double-degenerated LUMO belonging to the irreducible E symmetry representation. Excited states were computed using the Tamm-Dancoff approximation (TDA-DFT),[6] which predicts the two lowest-energy singlet electronic transitions ($S_0 \rightarrow S_1$ and $S_0 \rightarrow S_2$) degenerate in energy at 2.95 eV (Table S1) and mainly described by single HOMO → $LUMO_1$ and HOMO → $LUMO_2$ monoexcitations. The

energy predicted in gas phase for the $S_0 \rightarrow S_1$ and $S_0 \rightarrow S_2$ transitions is in good agreement with the experimental absorption band observed at 2.70 eV.[7] The excited states of the NHT monomer were also calculated using the OT-ωB97XD functional, which also predict the two lowest-energy $S_0 \rightarrow S_1$ and $S_0 \rightarrow S_2$ electronic transitions to be degenerate in energy, and to be bright with perpendicularly oriented transition dipole moments. However, OT-ωB97XD significantly overestimates these $S_0 \rightarrow S_1$ and $S_0 \rightarrow S_2$ excitations (~3.45 eV) compared to the experimental values. Therefore, the predicted energy values for the $S_0 \rightarrow S_1$ and $S_0 \rightarrow S_2$ transitions computed at B3LYP-D3 are used as E (FE-type excitation) in Eq. S3.

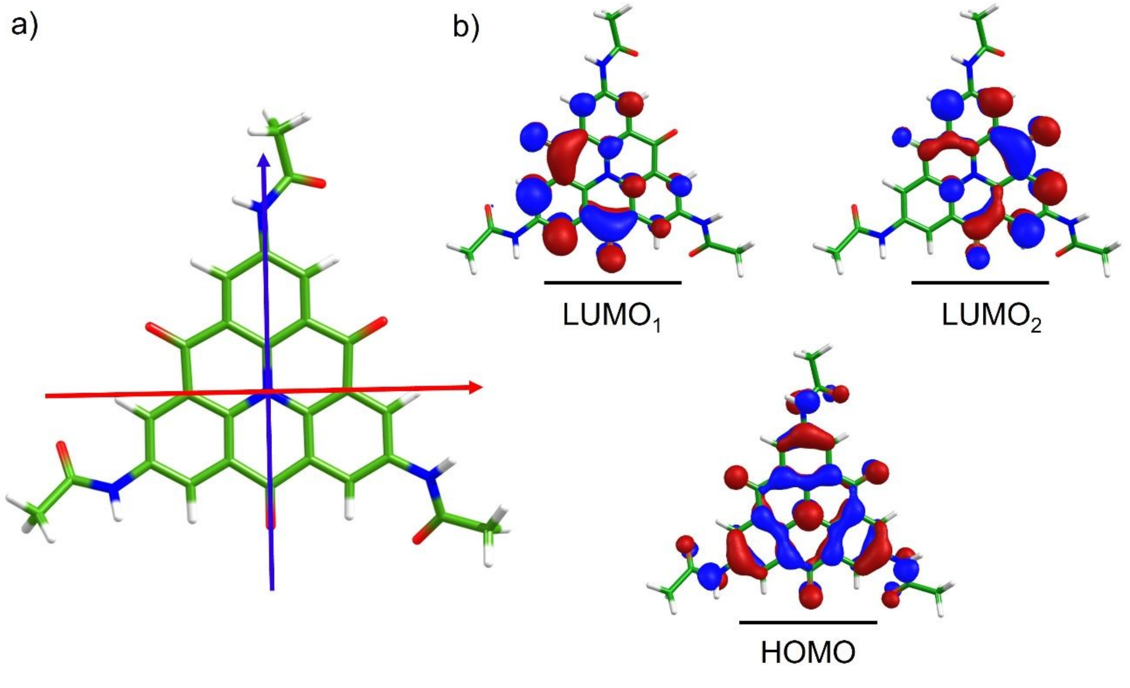

Figure S3. a) Optimized structure of a *N*-heterotriangulene derivative bearing acetamide peripheral groups. Blue and red arrows represent the direction of the transition dipole moment of the S_1 and S_2 states, respectively. b) Representation of the HOMO, $LUMO_1$ and $LUMO_2$ of the NHT derivative.

Table S1. Excitation energy (ΔE), components of the transition dipole moment (μ), and oscillator strength (f) calculated at the TDA-DFT B3LYP-D3/6–31G** level for the lowest-lying excited states of the NHT monomer.

State	ΔE (eV)	μ_x (a.u)	μ_y (a.u)	μ_z (a.u)	f
S_1	2.95	0.780	1.045	0.000	0.1232
S_2	2.95	-1.045	0.780	0.000	0.1232

As explained in the main text, ideal dimers were built up from the central part of the previously-optimized pentamers, at the B3LYP-D3/6-31G** level, to avoid terminal structural effects. Likewise, an extra ideal dimer (C_3-symmetry) was constructed where the amide groups are kept in the molecular plane of the π-conjugated core being not able to form intermolecular H-bonds (Figure S4). The low-lying singlet excited states of the dimer models were computed within the TDA-DFT approach using both the B3LYP-D3 and OT-ωB97XD functional.

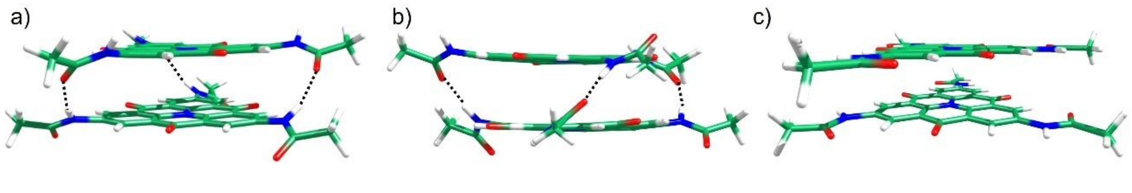

Figure S4. Representation of the three ideal dimers built based on models I and II and use for further parameterization of the model Hamiltonians: a) H-bonded C_3-symmetry dimer, b) H-bonded C_1-symmetry dimer and c) non-H-bonded C_3-symmetry dimer.

For the dimers, hereafter denoted as AB, both density functionals provide a similar picture (Table S2), where the lowest singlet excited states (S_1 and S_2) exhibit a remarkable CT character (A^-B^+ states), and are followed at higher energies by two pairs of FE-type A^*B and AB^* excited states (S_3/S_4 and S_9/S_{10}) and two CT A^+B^- states (S_{13}/S_{14}). A more detailed analysis of the data reported in Table S2 reveals some significant differences

between the B3LYP-D3 and OT-ωB97XD results, especially as what concerns the energy gap between the adiabatic FE-mostly and CT-mostly S_1/S_2 states (ΔE_{FE-CT}). This energy difference is reduced from 0.67 and 0.95 eV at the B3LYP-D3 level to 0.43 and 0.73 eV at the OT-ωB97XD level. Therefore, in the following, we will use B3LYP to estimate parameters obtained from single-molecule calculations, and OT-ωB97XD to compute the parameters for the dimers as the ΔE_{FE-CT} energy gaps and electronic and excitonic couplings.

Table S2. Vertical excitation energy (ΔE) calculated for selected adiabatic singlet excited states of the ideal NHT dimers at the DFT level using the B3LYP-D3 and OT-ωB97XD (ω = 0.15 Bohr^{-1}) functionals. The selected adiabatic excited states are those that can be easily associated with quasi-diabatic states of FE-type (A^*B and AB^*) and CT-type (A^+B^- and A^-B^+) nature.

H-bonded C_3-symmetry dimer					
B3LYP/6-31G**			OT-ωB97XD/6-31G**		
States	ΔE (eV)	Nature	States	ΔE (eV)	Nature
S_1, S_2	2.16	A^-B^+	S_1, S_2	2.92	A^-B^+
S_3, S_4	2.83	A^*B	S_3, S_4	3.35	A^*B
S_9, S_{10}	3.11	AB^*	S_9, S_{10}	3.65	AB^*
S_{28}, S_{29}	3.50	A^+B^-	S_{18}, S_{19}	4.24	A^+B^-
H-bonded C_1-symmetry dimer					
B3LYP/6-31G**			OT-ωB97XD/6-31G**		
States	ΔE (eV)	Nature	States	ΔE (eV)	Nature
S_1, S_2	2.33 / 2.54	A^-B^+	S_1, S_2	3.03 / 3.17	A^-B^+
S_3, S_4	2.63 / 2.74	A^*B & AB^*	S_3, S_4	3.33 / 3.40	A^*B
S_5 S_6	2.85 / 2.91	A^*B & AB^*	S_5, S_6	3.46 / 3.55	AB^*
S_7, S_8	2.97 / 3.02	A^+B^-	S_{11}, S_{14}	3.66 / 3.91	A^+B^-
Non H-bonded C_3-symmetry dimer					
B3LYP/6-31G**			OT-ωB97XD/6-31G**		
States	ΔE (eV)	Nature	States	ΔE (eV)	Nature
S_1, S_2	2.49	A^-B^+ & A^+B	S_1, S_2	3.11	A^-B^+ & A^+B^- A^*B & AB^*
S_3, S_4	2.70	A^-B^+ & A^+B	S_3, S_4	3.31	A^*B & AB^*
S_5, S_6	3.02	A^*B & AB^*	S_9, S_{10}	3.68	A^-B^+ & A^+B^-
S_7, S_8	3.20	A^*B & AB^*	S_{13}, S_{14}	3.86	A^-B^+ & A^+B^- A^*B & AB^*

It is noteworthy that the H-bonded supramolecular arrangement in the C_3-symmetry dimer causes an energy splitting between the two pairs of CT excitations (A^+B^- and A^-B^+). This lifting of degeneracy is due to the electrostatic stabilization (destabilization) of the CT excitations that are polarized parallel (antiparallel) to the electric field generated by the amide groups. As a matter of fact, excited-state degeneracy is recovered when either increasing the intermolecular distance *d* between the planes of the two molecules constituting the H-bonded dimer (Figure S5) and, particularly, when forcing the amide groups to remain in the molecular plane, thus effectively breaking the triple H-bond array (Figure S6). Figure S5 clearly reveals that the CT states (A^+B^- and A^-B^+) shift to higher energies when increasing the intermolecular distance (in line with Mulliken's law). Simultaneously, a reduction of the energy splitting between the A^+B^- and A^-B^+ states takes place owing to the smaller interaction of the holes and electrons with the permanent dipole moment of the other molecule. Although the analysis of the excited states is more difficult for the purely π-stacked dimer with no H-bonds due to the delocalized nature of the molecular orbitals involved in the excitations, Figure S6 shows a set of CT-type excited states whose energy increases with the intermolecular distance and are now degenerate in energy. These findings highlight how the orientation of the amide groups tune the permanent dipole moment within the supramolecular arrangement and, consequently, modulate the relevant $\Delta E_{FE\text{-}CT}$ energy gap. The three dimer models (Figure S4) are used to delve into how the energy position of CT states influences the optical and excitonic properties of the NHT-based supramolecular polymer (*vide supra*).

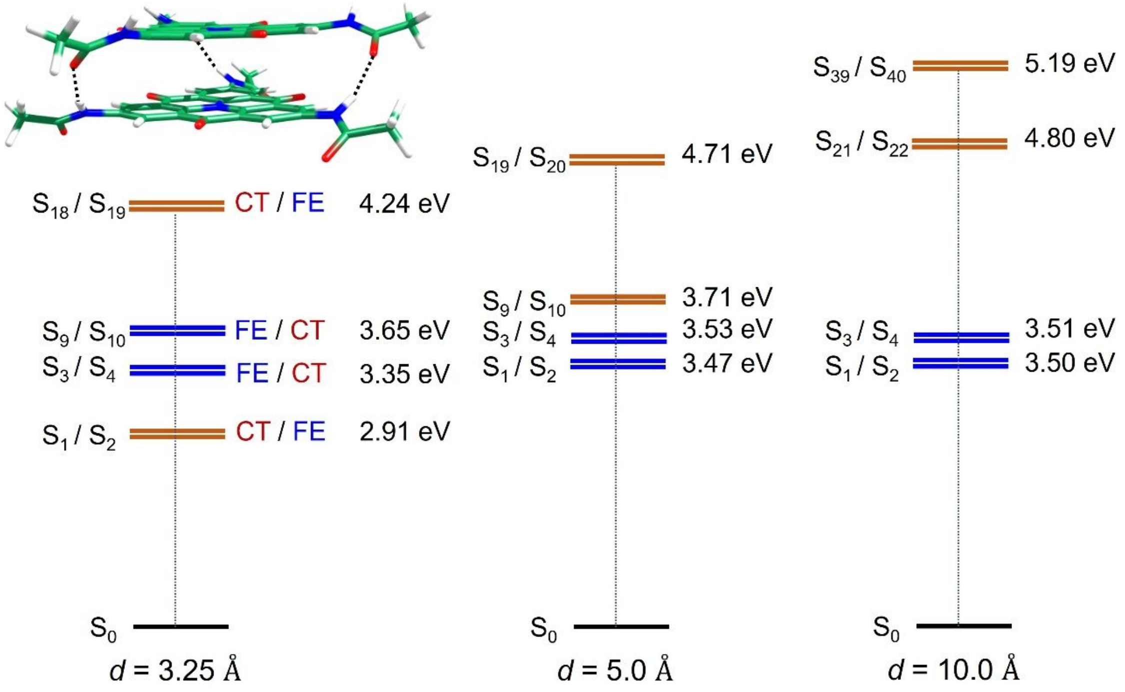

Figure S5. Energy diagram of the low-lying FE and CT states of the C_3-symmetry H-bonded NHT dimer at different intermolecular distances (*d*). A representation of the dimer with an intermolecular distance of 3.25 Å is provided.

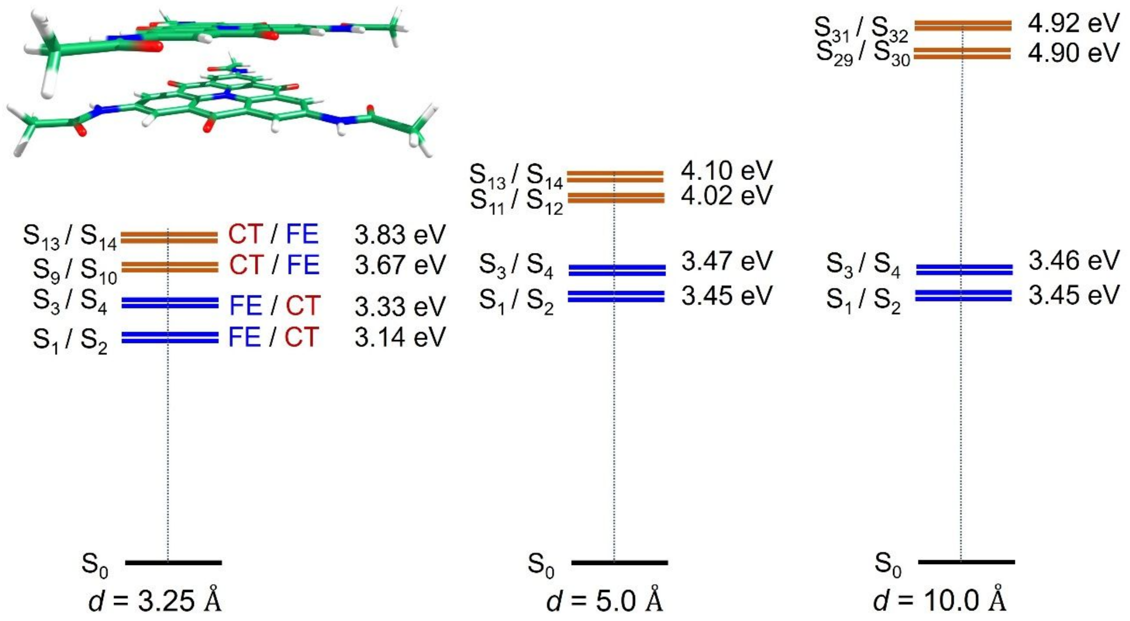

Figure S6. Energy diagram of the low-lying FE and CT states of the C_3-symmetry non-H-bonded dimer at different intermolecular distances (*d*). A representation of the dimer with an intermolecular distance of 3.25 Å is provided.

S5 Diabatic model Hamiltonian

Table S3 displays the FE and CT excitation energies (ΔE_{FE} and ΔE_{CT}), the excitonic couplings (J) and the hole and electron transfer integrals (t_h and t_e) computed for the three different dimers with decreasing dipole moment along the stacking direction: H-bonded C_3, H-bonded C_1, and non-H-bonded C_3 dimers. The energy parameters given in Table S3 were estimated within the FPHD diabatization protocol using the results obtained for the excited states of the dimers at the TDA-DFT OT-ωB97XD/6-31G** level. On one hand, the FE-type diabatic states are found for the three dimer models in a narrow energy window between 3.40 and 3.55 eV. However, as anticipated from the analysis of the adiabatic states, the energetic position of the CT states changes much more along the different dimer models. In particular, the CT states are centered around 3.55 eV for the non-H-bonded C_3-symmetry apolar dimer. When a dipole moment due to the amide groups orientation is introduced as a consequence of H-bond formation, an energy splitting between the A^+B^- and A^-B^+ diabatic states appears, which is significantly higher for the H-bonded C_3 dimer (1.12 eV) than for the H-bonded C_1 dimer (maximum 0.62 eV) due to the higher dipole moment of the former. On the other hand, the values calculated for the couplings J, t_h, and t_e are in the same range without being affected by the increase or decrease of the dipole moment. Therefore, to check specifically the influence of the $\Delta E_{FE\text{-}CT}$ energy offset, the couplings obtained for the C_3-symmetry dimer are used in the following simulations (Table S4).

Table S3. Diabatic energies and couplings obtained at the OT-ωB97XD level by using the FPHD diabatization scheme for three different dimer models. *x* and *y* subscripts are used to describe the main orientation of the transition dipole moment to distinguish between degenerate states. A schematic picture of a C_3-symmetry dimer, with molecules A and B and the direction of the local (red arrows) and total (black arrow) dipole moments (chemist direction convention), is provided.

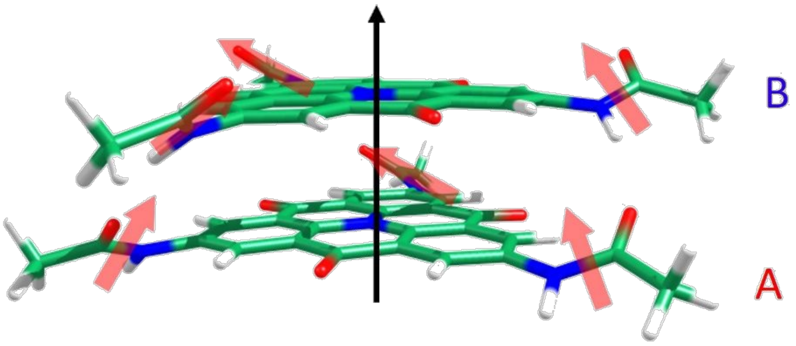

Parameter	H-bonded C_3	H-bonded C_1	non-H-bonded C_3
E_{A^*B} (eV)	3.527	3.41/3.50	3.460
E_{AB^*} (eV)	3.423	3.39/3.50	3.400
$E_{A^+B^-}$ (eV)	4.168	3.57/3.91	3.613
$E_{A^-B^+}$ (eV)	3.052	3.29/3.51	3.502
$J^{xx} = J^{yy}$ (meV)	49.3	32.1/37.5	38.8
$J^{xy} = -J^{yx}$ (meV)	15.2	33.8/20.7	40.9
$t_e^{xx} = t_e^{yy}$ (meV)	78.0	101.0/89.4	63.6
$t_e^{xy} = -t_e^{yx}$ (meV)	7.9	12.9/52.4	60.5
$t_h^{xx} = t_h^{yy}$ (meV)	–238.7	–173.7/–167.9	–251.7
$t_h^{xy} = -t_h^{yx}$ (meV)	31.4	73.2/20.3	33.3

Another important aspect of our model is that the states of the same nature degenerate in energy are condensed in a single effective state for the modelling of the optical and excitonic properties. A single effective state model is generally used to simplify a complex situation when multiple states degenerate in energy are present (as it is the case here for the NHT-based system). The main idea is based on establishing an effective

coupling between two states that would have the same electron/exciton transfer rate constant as a global rate constant involving multiple electron/exciton transfer processes (from one state at one site to different states degenerate in energy at another site). This is a common approach to achieve a simplified picture in the field of charge transport.[8–10] Table S4 contains the effective couplings that have been finally used in the model together with the energies for the different states (E, E_{+-} and E_{-+}). The sign of the effective coupling is set to coincide with the sign of the highest direct coupling (Table S4).

Table S4. Set of parameters used for the subsequent modelling of the optical and excitonic bands in the different situations.

	H-bonded C_3	H-bonded C_1	non-H-bonded C_3
E (eV)	2.88	2.88	2.88
E_{+-} (eV)	2.18	2.78	2.98
E_{-+} (eV)	3.28	3.07	2.98
J^{eff} (meV)	51.6	51.6	51.6
$t_{\mathrm{e}}^{\mathrm{eff}}$ (meV)	78.4	78.4	78.4
$t_{\mathrm{h}}^{\mathrm{eff}}$ (meV)	–240.8	–240.8	–240.8

Effective couplings were computed as $Z^{eff} = sign(Z^{xx})\sqrt{\left(Z^{xx}\right)^2 + \left(Z^{xy}\right)^2}$ where Z is either J, t_{h}, or t_{e}.

Relaxation energies of the excited, cationic, and anionic states of the central NHT unit, with no amide substituents, were estimated as the difference between the energy of the corresponding state in the minimum of the ground state and the optimized geometry of the corresponding adiabatic potential energy surface, obtaining values of 0.072, 0.260, and 0.082 eV for the S_1 excited singlet, the cation, and the anion, respectively. Thus, to include the vibronic progression in the simulation of the optical properties, an effective vibration ($\hbar\omega_0 = 1200$ cm^{-1}) that couples with the excitation was estimated from the experimental absorption spectrum of the diluted molecule.[11] The corresponding Huang-

Rhys factors (S) were estimated as $S_i = \lambda_i / \hbar\omega$, ($S_*$, S_+, and S_-) obtaining values of: 0.481, 1.751, and 0.549 for S_*, S_+, and S_-, respectively.

S6 Simulation of absorption and emission spectra of NHT-based systems

Figure S7 displays the absorption and emission spectra simulated for the single NHT monomer, where the 0–0 transition energy (ΔE_{0-0}) was set at 2.88 eV based on the energy difference between the vertical $S_0 \rightarrow S_1$ electronic transition (2.95 eV) and the relaxation energy of the S_1 excited state (0.072 eV). The proposed model provides a good agreement with respect to the experimental spectra, especially for the relative intensity of the peaks defining the vibrational progression.[11] The model used to calculate the spectra of the NHT monomer shows no Stokes shift since solvent effects, which are responsible for Stokes shifts in single rigid molecules, have not been included. Nevertheless, the model is able to account for the Stokes shift resulting from the supramolecular aggregation in the dimer, which is more relevant in this context.

Regarding the transition dipole moments for the FE states ($|n\rangle$), we employed the transition dipole moment of the isolated molecule for the excited state S1 (Table S1) for the first FE electronic excitation (site 1). For the rest of the FE excitations, the transition dipole moment was rotated along the z axis according to the $(n-1)\cdot 36^{\circ}$, where n corresponds to the site number in Eq. S3. The CT states were assumed to be completely dark and, therefore, the transition dipole moments are set to be 0.

To compute the optical properties of the aggregates (k = 0), we need 10 molecular sites to get a full helical pitch. This gives rise to 30 electronic states, 10 Frenkel states and 20 nearest neighbor CTs including those necessary due to the periodic boundary conditions. Likewise, we use a basis cutoff with the maximum number of vibrational excited quanta

limited to be 5. Therefore, the size of the one-particle basis is $N_{1p} = N_{FE} \cdot (v_{\max} + 1) = 60$, whereas the number of two-particle basis functions is defined as $N_{2p} = N_{FE} \cdot (N_{FE} - 1) \cdot \sum_{i=1}^{v_{\max}} i = 1350$. Note that the two-particle functions for all the molecular pairs are not restricted to nearest neighbors. Finally, the number of nearest neighbor CT basis functions can be estimated as $N_{CT\_basis} = N_{CT} \cdot \sum_{i=1}^{v_{\max}+1} i = 420$, which makes a total of 1830 basis functions.

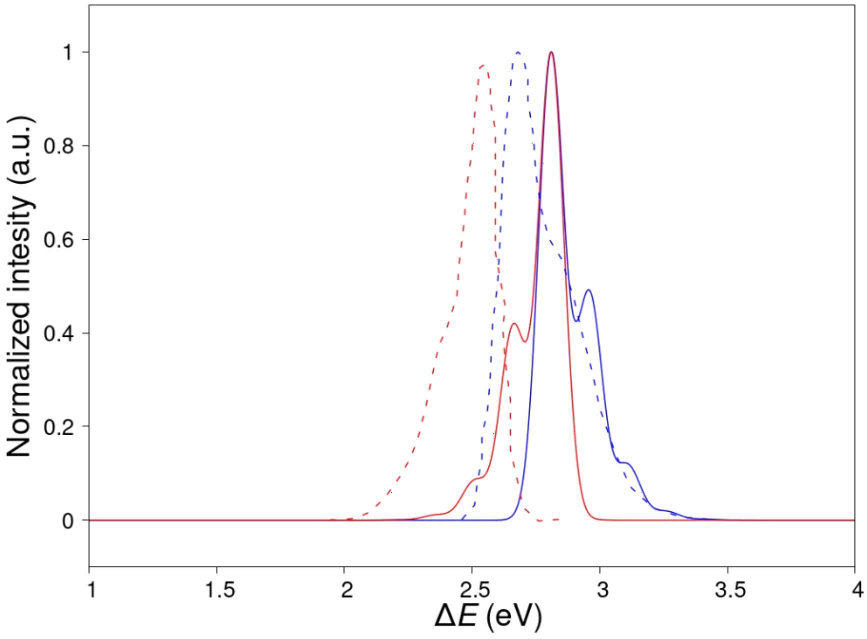

Figure S7. Normalized absorption (blue) and emission (red) spectra experimentally registered in THF solution for NHT-based monomers (dashed lines) and computationally simulated (solid lines) using the FCTH Hamiltonian. Experimental spectra are readapted from ref. 11.

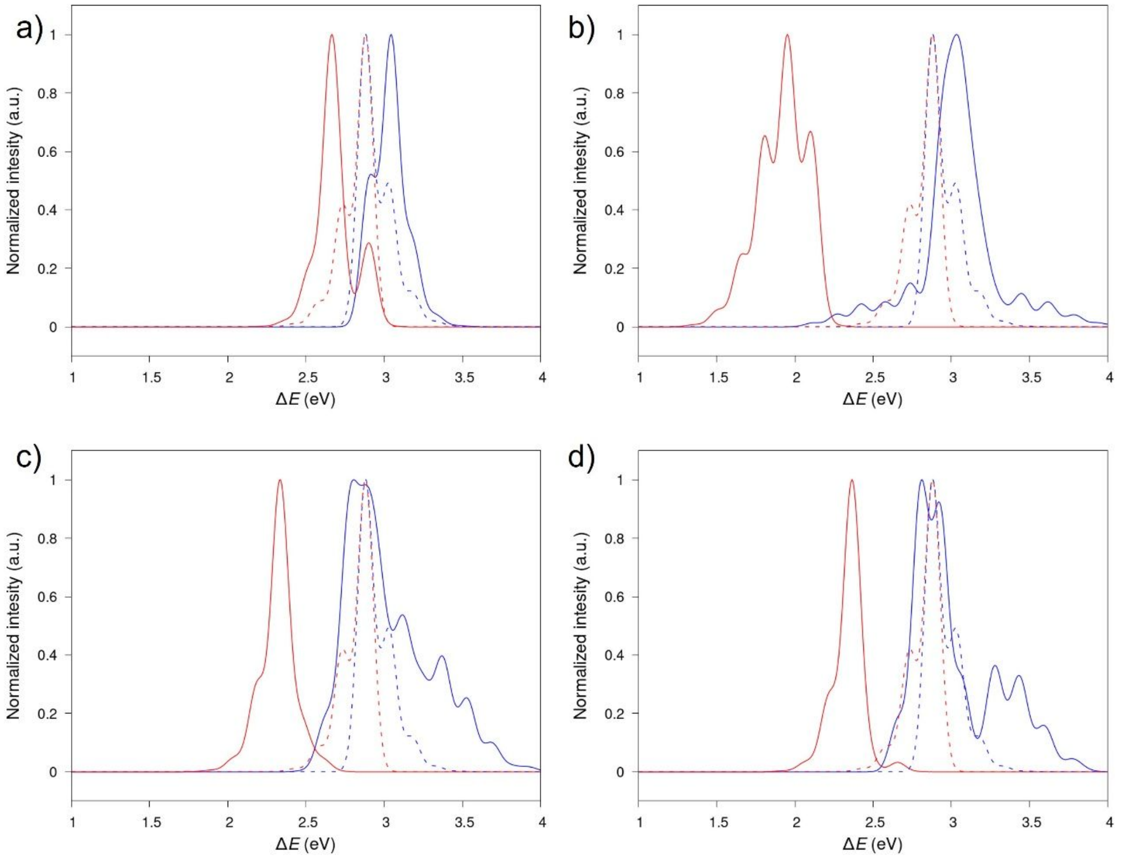

Figure S8. Simulated absorption (black) and emission (red) normalized spectra computed for the helical NHT aggregate using different models: a) H-bonded C_3-symmetry aggregate without including CT states, b) H-bonded C_3-symmetry aggregate, c) H-bonded C_1-symmetry aggregate, and d) non-H-bonded C_3-symmetry aggregate. Static disorder is not included in the simulation of these spectra. Dashed lines correspond to the monomer spectra as reference.

S7 Band structure and exciton transport properties for the model aggregates

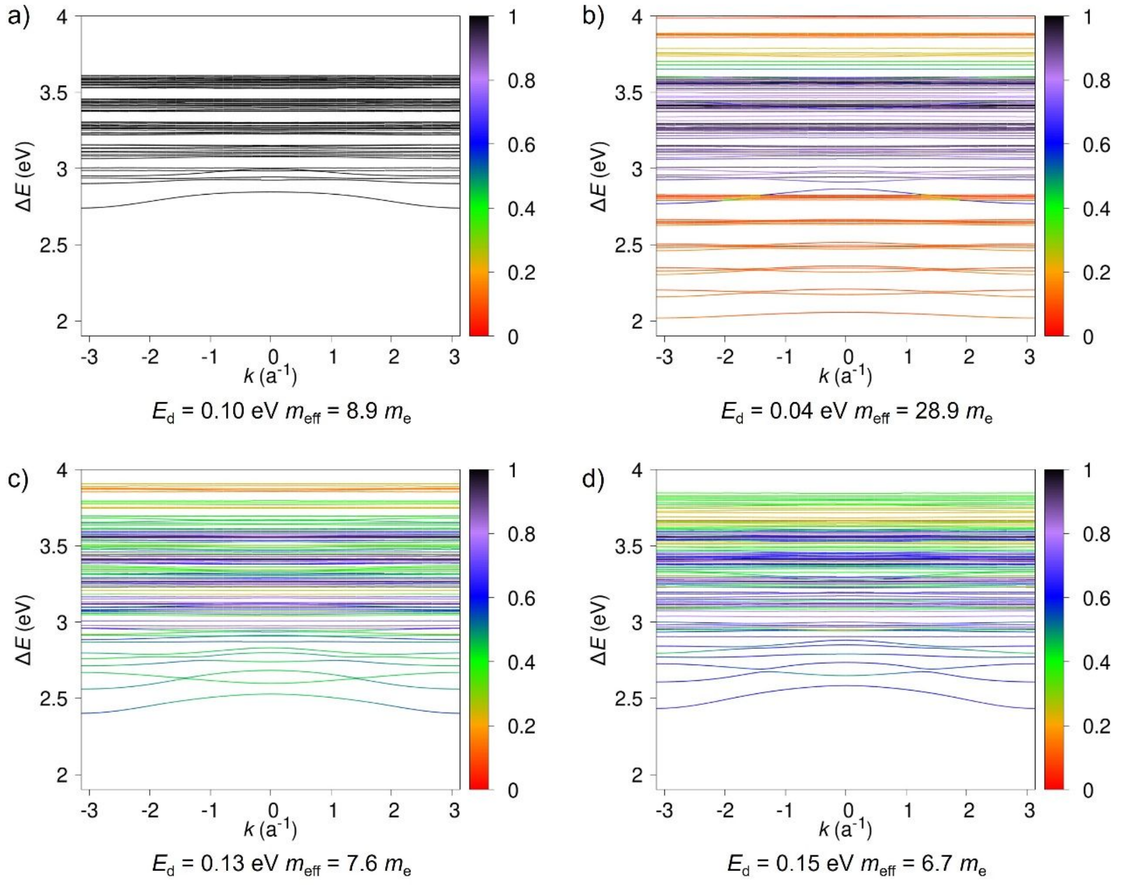

Figure S9. Band diagram of the vibronically dressed electronic states calculated for the helical NHT aggregate using different models: H-bonded C_3-symmetry polar model without including CT states (a), and H-bonded C_3-symmetry polar (b), H-bonded C_1-symmetry polar (c), and non-H-bonded C_3-symmetry apolar (d) models. Color scale represent the Frenkel character of the band at each *k*-point.

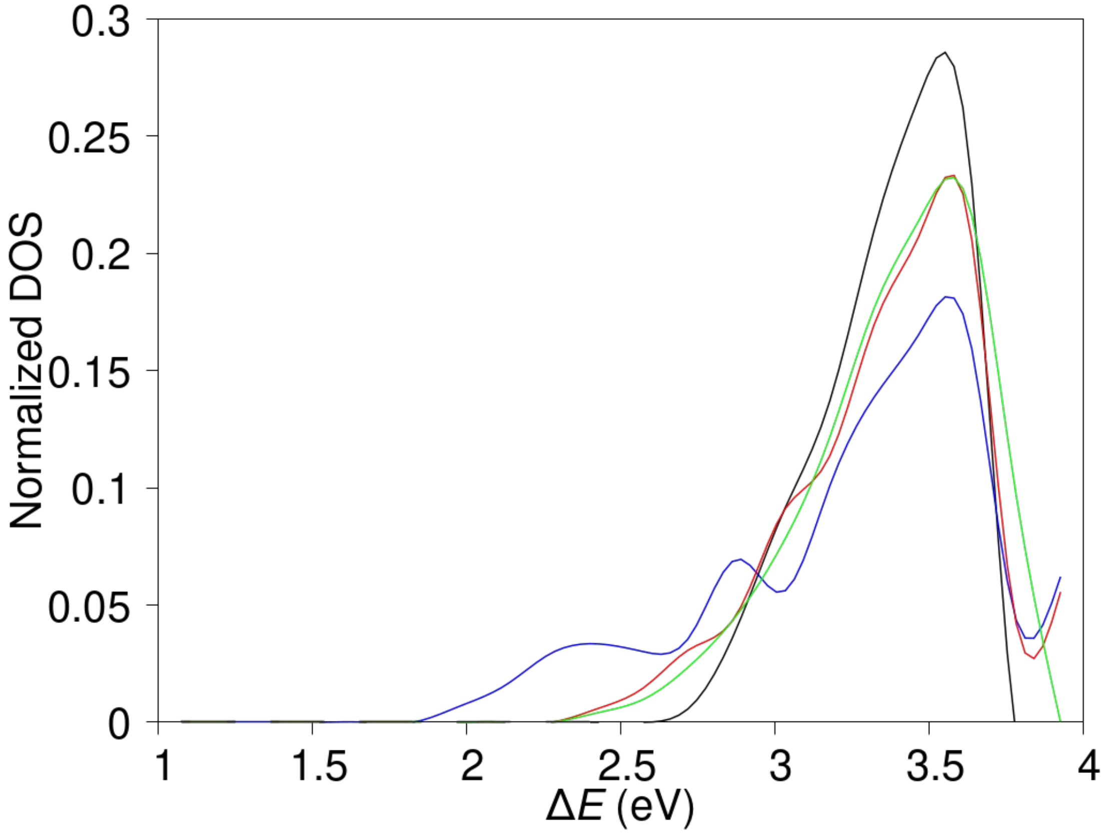

Figure S10. Normalized density of states computed from the vibronic band structure (Figure S9) for the helical NHT aggregate using different models: H-bonded C_3-symmetry polar model without including CT states (black), and H-bonded C_3-symmetry polar (blue), H-bonded C_1-symmetry polar (red), and non-H-bonded C_3-symmetry apolar (green) models.

S8 References

(1) Stein, T.; Kronik, L.; Baer, R. Reliable Prediction of Charge Transfer Excitations in Molecular Complexes Using Time-Dependent Density Functional Theory. *J. Am. Chem. Soc.* **2009**, *131*, 2818–2820.

(2) Chai, J. Da; Head-Gordon, M. Long-Range Corrected Hybrid Density Functionals with Damped Atom-Atom Dispersion Corrections. *Phys. Chem. Chem. Phys.* **2008**, *10*, 6615–6620.

(3) Francl, M. M.; Pietro, W. J.; Hehre, W. J.; Binkley, J. S.; Gordon, M. S.; DeFrees, D. J.; Pople, J. A. Self-consistent Molecular Orbital Methods. XXIII. A Polarization-type Basis Set for Second-row Elements. *J. Chem. Phys.* **1982**, *77*, 3654–3665.

(4) Wang, Y. C.; Feng, S.; Liang, W.; Zhao, Y. Electronic Couplings for Photoinduced Charge Transfer and Excitation Energy Transfer Based on Fragment Particle-Hole Densities. *J. Phys. Chem. Lett.* **2021**, *12*, 1032–1039.

(5) Cardoso, J. F.; Souloumiac, A. Jacobi Angles for Simultaneous Diagonalization. *SIAM J. Matrix Anal. Appl.* **1996**, *17*, 161–164.

(6) Hirata, S.; Head-Gordon, M. Time-Dependent Density Functional Theory within the Tamm–Dancoff Approximation. *Chem. Phys. Lett.* **1999**, *314*, 291–299.

(7) Haedler, A. T.; Kreger, K.; Issac, A.; Wittmann, B.; Kivala, M.; Hammer, N.; Köhler, J.; Schmidt, H.-W.; Hildner, R. Long-Range Energy Transport in Single Supramolecular Nanofibres at Room Temperature. *Nature* **2015**, *523*, 196–199.

(8) D'Avino, G.; Olivier, Y.; Muccioli, L.; Beljonne, D. Do Charges Delocalize over Multiple Molecules in Fullerene Derivatives? *J. Mater. Chem. C* **2016**, *4*, 3747–3756.

(9) Castet, F.; D'Avino, G.; Muccioli, L.; Cornil, J.; Beljonne, D. Charge Separation Energetics at Organic Heterojunctions: On the Role of Structural and Electrostatic Disorder. *Phys. Chem. Chem. Phys.* **2014**, *16*, 20279–20290.

(10) Liu, T.; Troisi, A. What Makes Fullerene Acceptors Special as Electron Acceptors in Organic Solar Cells and How to Replace Them. *Adv. Mater.* **2013**, *25*, 1038–1041.

(11) Wittmann, B.; Wenzel, F. A.; Wiesneth, S.; Haedler, A. T.; Drechsler, M.; Kreger, K.; Köhler, J.; Meijer, E. W.; Schmidt, H. W.; Hildner, R. Enhancing Long-Range Energy Transport in Supramolecular Architectures by Tailoring Coherence Properties. *J. Am. Chem. Soc.* **2020**, *142*, 8323–8330.
