# Supplementary material for: Optical Properties of H-Bonded Heterotriangulene Supramolecular Polymers: Charge-Transfer Excitations Matter
Source: J Phys Chem Lett. 2024 Jul 25;15(30):7814–21. doi: 10.1021/acs.jpclett.4c01520 (PMC11299171; doi:10.1021/acs.jpclett.4c01520)
Supplement: Supplementary file 2 — jz4c01520_si_002.pdf [file jz4c01520_si_002.pdf]

jz-2024-01520f.R1

Name: Peer Review Information for "Optical Properties of H-bonded Heterotriangulene Supramolecular Polymers: Charge-Transfer Excitations Matter"

First Round of Reviewer Comments

Reviewer: 1

Comments to the Author

In this paper the authors examine the effect of Charge-Transfer on optical properties in triangulate fibrils. The authors conclude that including CT states strongly improve the agreement with experiment. While the findings are plausible a lot of information is hidden in the SI and not clearly enough written to be reproduced. The impact of the paper is rather specialised and I think that reshaping it in a more clearly written JPC A paper without continuous references to important findings in the SI would be the best option.

Saikin is mentioned in the text, but no actual reference is given.

I assume that the authors refer to 10.1021/acs.jpcc.7b08933, but this is inappropriate.

Hildner and co-workes also recently published a paper on modelling the long range transport in trianguline fibrils. doi.org/10.1021/acs.jpclett.3c03586

I miss more information on the disorder model. The description on page S7 is rather hard to follow. I am in doubt if each site has a different energy or not and it not specific what the average energy values are. How was the static disorder chosen and why is it the same for Frenkel and CT states? The Huang-Rhys factors are different for the states so why not the disorder?

Why is the applied model underestimating the monomer stokes shift and overestimating the vibronic progressions?

How were the band diagrams of Figure 5 obtained?

It would be useful to have one single Table where the parameters used in each of the models is given explicitly. Now the symbols used in Tables S3 and S4 are different from those used in section S3. Furhtermore, the tables have /-signs which makes it unclear which value was actually used.

How many basis functions in total in the Frenkel-CT model?

For Eq. S12 What is nu, omega and omega_0? How is the radiative lifetime independent on omega and nu? Is this equation really correct?

The single effective state model is insufficiently described. Where does the equation for the effective couplings come from? How is the sign of the effective coupling chosen?

The value of \omega_0 is never defined. Maybe it is 1200 cm-1 guessing that this the same as the definition of \omega on page S18.

It would be informative to compare the density of states with the optical spectra.

It is not transparent how the transition dipole moments for the exciton and CT states are defined in the presented models.

Reviewer: 2

Comments to the Author

The manuscript is very well written and logically developed, concerning an important topic in chemical physics - that of energy transfer in organic materials. The authors analyze theoretically the steady-state absorption and emission spectra as well as the exciton band structure of the supramolecular "polymer" consisting of a non-covalently bound linear array of triangulene chromophores. They employ a Frankel - CT Holstein Hamiltonian, with ground-state geometries and parameters derived mainly from DFT theory. Overall they obtain very good agreement with the experimentally measured spectra and show that by including the CT states, the exciton effective mass can be substantially lowered, underscoring the importance of Frenkel-CT mixing in exciton transport. The paper will appeal to a large cohort of researchers in the organic materials field and should be published after addressing some minor points:

1) Can the authors clarify what they mean by a flipped amide array? Perhaps with the help of a slightly modified figure? Also why does the flipped array have a smaller dipole moment?

2) The energy level ordering of the CT states and Frenkel states should be better clarified. Why is the A-B+ CT state lowest in energy? Apparently this has to do with interactions with the permanent dipole moment established by the amide arrays. Does this mean that if the ground state dipole moment is pointing "up" then the CT states which align in the downward direction (with the cation below the anion) are lower in energy?

3) Can the authors define the electron and hole transfer integrals, te and th? This is important, especially for the signs. For example, the hole integral th is the negative of the Hamiltonian matrix element connecting the neighboring HOMO levels (so th=-tH), whereas te is directly the matrix element connecting the neighboring LUMO levels. The negative sign in th arises because moving a hole forward is the same as moving a HOMO electron backwards. (see Gisslen and Scholz, PRB 2009) In this notation, Eq. S5 should be written with a positive sign in front of the second term in brackets. Perhaps the authors defined the hole transfer integral without the negative sign, so that the negative sign in Eq.S5 is then appropriate? In any case, definitions of te and th will resolve any inconsistency. (Also of importance is how the phase of the orbitals is chosen in the first place!)

4) Can the authors also report the HR factor needed to obtain agreement with the monomer spectrum (bottom of page 7).

5) Some simple spelling: pg 4 last paragraph "tunned" also "Becke Jonhson"

Author's Response to Peer Review Comments:

Valencia, July 2nd 2024

Dear Editor,

Please find attached a **revised version** of the manuscript entitled "*On the Optical Properties of H-bonded Heterotriangulene Supramolecular Polymers: Charge-Transfer Excitations Matter*" by J. Cerdá, E. Ortí, D. Beljonne and J. Aragó (**jz-2024-01520f**), to be considered for publication as a full paper in *The Journal of Physical Chemistry Letters*.

We are pleased that the Reviewers and also the Editor have considered our manuscript of potential interest to the readers of *The Journal of Physical Chemistry Letters* even though it requires a revision of some technical aspects. We have taken into account the reviewers' comments (see the **point-by-point response**) and the manuscript has been accordingly corrected. All corrections in the revised manuscript and Supporting Information have been marked in yellow and these marked files have been uploaded in the "Supporting Information for Review Only" section. After considering all the points addressed by the Reviewers, we hope the article now fulfils the requirements to be accepted.

I confirm that all authors approve this submission and that the manuscript has not been published elsewhere and is not under consideration by another journal.

Looking forward to hearing from you at your earliest convenience.

Yours sincerely,

Dr. Juan Aragó
Instituto de Ciencia Molecular (ICMol)
Universitat de València
E-mail: juan.arago@uv.es
Tel.: +34 963543154

Point-by-point reply to Reviewers

Reviewer: 1

Recommendation: Reconsider as an article in The Journal of Physical Chemistry A/B/C.

In this paper the authors examine the effect of Charge-Transfer on optical properties in triangulate fibrils. The authors conclude that including CT states strongly improve the agreement with experiment. While the findings are plausible a lot of information is hidden in the SI and not clearly enough written to be reproduced. The impact of the paper is rather specialised and I think that reshaping it in a more clearly written JPC A paper without continuous references to important findings in the SI would be the best option.

We appreciate the reviewer's criticisms regarding the clarity and reproducibility of our work in its current state. By addressing all the points raised by the reviewer, we are confident that both the revised version of the main manuscript and the Supporting Information have significantly gained in clarity and reproducibility.

On the other hand, and with all due respect to the Reviewer, we disagree with the assessment of the impact of our work considered to be rather specialized. In this regard, our model shows the relevance of charge-transfer (CT) excitations to shape the optical properties and potentially improve the exciton migration in *N*-heterotriangulene (NHT) supramolecular polymers. This idea is not widely extended in the Physical Chemistry and Material Chemistry communities. Additionally, our study also reveals that the employed Hamiltonian (a Frenkel-CT Holstein Hamiltonian), after experimental validation, is the minimum model to study the exciton transport, which has not been considered so far in NHT-based molecular aggregates.

Saikin is mentioned in the text, but no actual reference is given. I assume that the authors refer to 10.1021/acs.jpcc.7b08933, but this is inappropriate.

We totally agree with the reviewer that is inappropriate to mention the work of Saikin and coworkers and not to cite it. This was an oversight and we thought the work was properly cited. We have corrected our mistake and the article is now correctly cited (Page 3).

Hildner and co-workes also recently published a paper on modelling the long range

transport in trianguline fibrils. doi.org/10.1021/acs.jpclett.3c03586

We thank the reviewer for pointing out this recent paper of Hildner and coworkers that we did not know in the moment of the submission. We have added a brief discussion in the introduction (Page 3) to properly cite that paper and discuss the main findings in the context of our work.

I miss more information on the disorder model. The description on page S7 is rather hard to follow. I am in doubt if each site has a different energy or not and it not specific what the average energy values are. How was the static disorder chosen and why is it the same for Frenkel and CT states? The Huang-Rhys factors are different for the states so why not the disorder?

In line with the reviewer's comment, we have slightly extended the description of how static disorder is introduced in our model (Pages S7 and S8 in the Supporting Information) to clarify further this aspect and avoid potential misinterpretations. Likewise, we respond below the points raised by the reviewer.

In our model, we incorporate the static disorder by selecting random values of all Frenkel and CT excitation energies according to a Gaussian distribution in a non-correlated manner. Therefore, all excitation energies are likely to be different within each realization and in between due to the lack of correlation during the independent selection of the diagonal energy values in the model Hamiltonian. The excitation energies used as mean values for the Gaussian distributions are now explicitly provided in Table S4 instead of the $\Delta E_{\text{FE-CT}}$ energy gap (previous version) that was less clear.

Regarding the chosen static disorder, we selected a standard deviation value of 130 meV according to Hildner and coworkers (J. Am. Chem. Soc. 2020, 142, 8323) that experimentally estimated the static disorder from spectral measurements for similar supramolecular polymers. Note that the introduction of static disorder only contributes to the total broadening of the spectra (Figure 3 and S8) without altering the Stokes shift. Nevertheless, we agree with the reviewer that the same magnitude of the static disorder for both type of excitations (Frenkel and CT) cannot be totally justified. In this regard, we have computed the absorption and emission spectra (see Figure 1 included here following) with different values of static disorder (65, 130 and 260 meV) for the CT excitations while the static disorder for the Frenkel states is kept to be constant (σ = 130 meV). The predicted spectra are quite similar, except for a slight broadening and, consequently, this decision (same static disorder for both excitations) is reasonable.

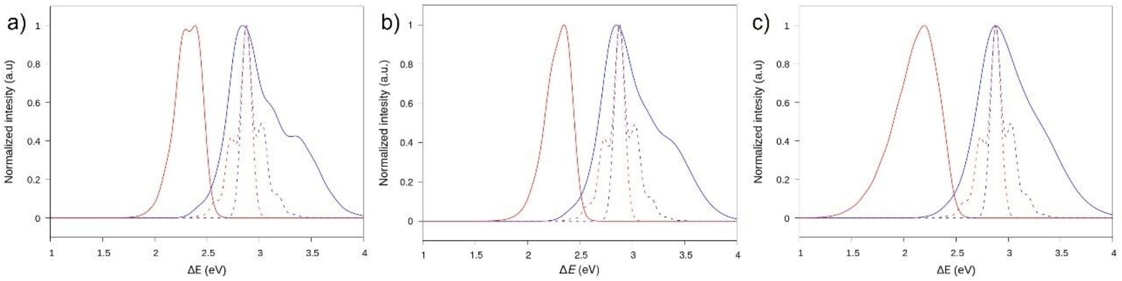

Figure 1. Simulated absorption and emission spectra for the helical H-bonded C_1-symmetry supramolecular polymer with a static disorder for the Frenkel excitations of 130 meV and different values for the CT excitations: 65 (a), 130 (b) and 260 meV (c).

Why is the applied model underestimating the monomer stokes shift and overestimating the vibronic progressions?

The applied model does not account for the Stokes shift on the monomer since we are using the same transition energy (ΔE_{0-0}) for absorption and emission. This was already explained in Page S19: "*The model used to calculate the spectra of the NHT monomer shows no Stokes shift since solvent effects, which are responsible for Stokes shifts in single rigid molecules, have not been included.*"

Regarding the differences between the experimental and simulated vibronic progressions for the monomer, they come exclusively from the Huang–Rhys factor. In our simulation, the Huang–Rhys factor is estimated from the relaxation energy computed by DFT and TDA-DFT calculations. These relaxations energies are quite small (~72 meV) and, consequently, the effective Huang–Rhys factor is also small (0.481). Nevertheless, we believe that, despite the lack of monomer Stokes shift, the agreement between the shape of the experimental and simulated spectra of the monomer is generally quite good.

How were the band diagrams of Figure 5 obtained?

The band diagrams of Figure 5 and Figure S9 are obtained though full diagonalization of the model Hamiltonian detailed from Eq. S2 to Eq. S6 within the basis set specified in Eq. S7 for each k point value. In particular, the band diagram in Figure 5 is a pure electronic picture where no vibrational levels were included and therefore the Huang–Rhys factors were set to 0 to avoid vibrational screening of the excitonic/electronic couplings (due to the Franck–Condon integrals) in the pure electronic picture.

It would be useful to have one single Table where the parameters used in each of the models is given explicitly. Now the symbols used in Tables S3 and S4 are different from those used in section S3. Furhtermore, the tables have /-signs which makes it unclear which value was actually used.

According to the reviewer's suggestion, we have modified Table S4 to explicitly include all the parameters used in the model and use the same notation as the Hamiltonian (Eqs. S2-S6 in Section S3). Consequently, the "/–"signs are not necessary now and have been removed.

Table S3 is a bit different compared to Table S4. Table S3 shows all the raw values obtained from the diabatization process where there are more states and couplings than those used in the model (Table S4). For instance, the diabatic A^*B and AB^* excitations are degenerate in energy and there exist direct- and cross-coupling terms (labelled as $J^{xx} = J^{yy}$ and $J^{xy} = -J^{yx}$ in Table S3). Nevertheless, the model used only includes a single effective Frenkel state per site and, thus, a single coupling between sites. This is the reason why Table S3 and S4 are separated. As mentioned above, Table S4 is the one collecting all the parameters used in the model Hamiltonian.

How many basis functions in total in the Frenkel-CT model?

To specify the question raised by the reviewer, we have added the following paragraph in Pages S19-S20:

"To compute the optical properties of the aggregates (k = 0), we need 10 molecular sites to get a full helical pitch. This gives rise to 30 electronic states, 10 Frenkel states and 20 nearest neighbor CT states including those necessary due to the periodic boundary conditions. Likewise, we use a basis cutoff with the maximum number of vibrational excited quanta limited to 5. Therefore, the size of the one-particle basis is $N_{1p} = N_{FE} \cdot (v_{\max} + 1) = 60$*, whereas the number of two-particle basis functions is defined as* $N_{2p} = N_{FE} \cdot (N_{FE} - 1) \cdot \sum_{i=1}^{v_{\max}} i = 1350$*. Note that two-particle functions for all the molecular pairs are not restricted to nearest neighbors. Finally, the number of nearest neighbor CT basis functions can be estimated as* $N_{CT\_basis} = N_{CT} \cdot \sum_{i=1}^{v_{\max}+1} i = 420$*, which makes a total of 1830 basis functions."*

For Eq. S12 What is nu, omega and omega_0? How is the radiative lifetime independent on omega and nu? Is this equation really correct?

First of all, we should apologize for the confusing notation of Eq. S12. Now, we have tried to fix the notation inconsistencies in Pages S7-S8.

ν is the number of excited vibrational quanta in the ground state for a given emission line. ω_0 is the frequency of the effective normal mode as in Eq. S6 and in the emission intensity (Eq. S9). The previous ΔE_a magnitude has been modified to $\omega_{a,k=0}$ (the energy of the vibronic eigenstate) for consistency with all expressions (Eq. S8-S12). The radiative lifetime is not independent of $\omega_{a,k=0}$ and ν. It depends on the radiative decay rates (Eq. S11) and these rates (Eq. S12) depend on the emission intensity ($\omega_{a,k=0}$ and ν).

The expression in Eq. S12 is correct. The reviewer might see that Eq. S12 is similar to Eq. 1 in *J. Am. Chem. Soc.* 2020, 142, 41, 17782–17786 expressed only for a single electronic state. Our expression is simply an extension (a summation) to account for all emissive vibronic states weighted by the Boltzmann distribution, where the intensity of the electronic excitation (squared modulus of the transition dipole moment) is replaced by the vibronic emission intensity (Eq. S9) without considering the broadening. In our expression, the *f(n)* term of Eq. 1 in *J. Am. Chem. Soc.* 2020, 142, 41, 17782 is set to be 1 because we work in vacuum.

Eq. S12 is also similar to that reported in another article (Eq. 11 in *J. Comput. Chem.* 2023, 44, 626). This expression computes the rate in the same way as we do here using Eq. S12 but integrating over the emission spectrum intensity, which can be more suitable for our case (multiple emission vibronic lines are present). However, we did some tests with different broadenings (σ values) in the Gaussian line shape function, and we saw that the radiative constant rates with this expression changes with the spectrum broadening. To avoid that dependence, we opted, instead of an integration over the emission spectrum, for a discrete summation over the emission vibronic states before the Gaussian convolution.

The single effective state model is insufficiently described. Where does the equation for the effective couplings come from? How is the sign of the effective coupling chosen?

The single effective state model seeks to simplify a complex situation when multiple states degenerate in energy are present, as it is the case here for the NHT-based system. The main idea is based on establishing an effective coupling between two states that would have the same electron/exciton transfer rate constant as a global rate constant involving multiple electron/exciton transfer processes (from one state at one site to

different states degenerate in energy at another site). This is a common approach to achieve a simplified picture in the field of charge transport (i.e., J. Mater. Chem. C, 2016, 4, 3747–3756, Phys. Chem. Chem. Phys., 2014, 16, 20279–20290 or Adv. Mater. 2013, 25, 1038–1041). The sign of the effective coupling is set to coincide with the sign of the highest direct coupling (see expression in Table S4).

We added extra information and proper references in Page S17 and S18 where the single effective state model is mentioned.

The value of \omega_0 is never defined. Maybe it is 1200 cm-1 guessing that this the same as the definition of \omega on page S18.

The reviewer is right that the value of ω_0 was not defined. That has been corrected in Page S18 ($\hbar\omega_0 = 1200$ cm^{-1}).

It would be informative to compare the density of states with the optical spectra. It is not transparent how the transition dipole moments for the exciton and CT states are defined in the presented models.

Following the reviewer's comment, we have computed the density of states (DOS) for the four models (Figure S10) and added a new paragraph (Page 16) that compares the DOS obtained from the band structure with the simulated absorption spectra (Figure 3).

The reviewer is right that it was not specified how the transition dipole moments are defined in the model used. For the first FE electronic excitation (site 1), the transition dipole moment of the isolated molecule for the excited state S_1 (Table S1) was employed. For the rest of the FE excitations, the transition dipole moment was rotated along the z axis according to the $(n-1)\cdot 36^o$, where n corresponds to the site number in Eq. S3. The CT states were assumed to be completely dark and, therefore, the transition dipole moments were set to zero. These aspects are now explained in Page S19. Also, we have added an extra sentence in Page S7 to clarify how the adiabatic transition dipole moments are calculated.

Reviewer: 2

Recommendation: This paper is publishable subject to minor revisions noted. Further review is not needed.

The manuscript is very well written and logically developed, concerning an important topic in chemical physics - that of energy transfer in organic materials. The authors analyze theoretically the steady-state absorption and emission spectra as well as the exciton band structure of the supramolecular "polymer" consisting of a non-covalently bound linear array of triangulene chromophores. They employ a Frankel -CT Holstein Hamiltonian, with ground-state geometries and parameters derived mainly from DFT theory. Overall they obtain very good agreement with the experimentally measured spectra and show that by including the CT states, the exciton effective mass can be substantially lowered, underscoring the importance of Frenkel-CT mixing in exciton transport. The paper will appeal to a large cohort of researchers in the organic materials field and should be published after addressing some minor points:

First of all, we would like to thank Reviewer 2 for his/her positive comments concerning the scientific quality and relevance of our study.

1) Can the authors clarify what they mean by a flipped amide array? Perhaps with the help of a slightly modified figure? Also why does the flipped array have a smaller dipole moment?

In line with the reviewer's comment, we have modified Figure 2 to clarify what we mean by a flipped amide array. We have highlighted the intermolecular H-bonding network where the amides are inverted (flipped) compared to the other two H-bonding networks. Additionally, we have stressed the direction of the dipole moment (NH→CO direction according to the chemical convention) with three colored arrows in the terminal amides for one molecule for a better comprehension. An inversion of an amide array reduces the component of the dipole moment parallel to the π-stacking direction, which is the direction that mainly influences the energy of the CT states. An extra sentence has been added in Page 5 to explain this point.

2) The energy level ordering of the CT states and Frenkel states should be better clarified.

Why is the A-B+ CT state lowest in energy? Apparently this has to do with interactions with the permanent dipole moment established by the amide arrays. Does this mean that if the ground state dipole moment is pointing "up" then the CT states which align in the downward direction (with the cation below the anion) are lower in energy?

We appreciate the reviewer’s comment because it brings the necessity to clarify the convention used in the manuscript for the dipole moment direction to avoid potential misunderstandings. Accordingly, the convention employed (chemical convention) has been indicated in the caption of Table S3. Likewise, a picture has been added in Table S3 labeling molecule A and B as well as the direction of the local (amides) and total dipole moments for further clarification.

3) Can the authors define the electron and hole transfer integrals, te and th? This is important, especially for the signs. For example, the hole integral th is the negative of the Hamiltonian matrix element connecting the neighboring HOMO levels (so th=-tH), whereas te is directly the matrix element connecting the neighboring LUMO levels. The negative sign in th arises because moving a hole forward is the same as moving a HOMO electron backwards. (see Gisslen and Scholz, PRB 2009) In this notation, Eq. S5 should be written with a positive sign in front of the second term in brackets. Perhaps the authors defined the hole transfer integral without the negative sign, so that the negative sign in Eq.S5 is then appropriate? In any case, definitions of te and th will resolve any inconsistency. (Also of importance is how the phase of the orbitals is chosen in the first place!)

We thank the reviewer for highlighting this potential confusing point. Following his/her suggestion, explicit definitions for t_h and t_e as Hamiltonian matrix elements between diabatic states ($\left\langle A^{-}B^{+}\middle|\hat{H}\middle|A^{*}B\right\rangle$ and $\left\langle A^{+}B^{-}\middle|\hat{H}\middle|A^{*}B\right\rangle$, respectively) are now provided in Page S6 and, consequently, the sign “–” has been removed in Eq. S5. This is more convenient since we obtain all the excitonic/electronic couplings directly from an adiabatic-to-diabatic transformation over states.

Concerning the phase, as all the couplings are obtained from a diabatization process over states (not orbitals), it is not necessary to explicitly control the phase of the molecular orbitals (HOMO or LUMO) of the individual molecules. Nevertheless, we have confirmed, as a double-check, the sign of t_h and t_e with a projection method (Phys. Chem. Chem. Phys., 2010, 12, 11103) where the phase of the orbitals has been controlled. In particular, the phase of individual orbitals (HOMO or LUMO) of molecule B is the same as the phase of orbitals (HOMO or LUMO) of molecule A after a translation along the *z*

axis (aggregation direction) and rotation along this axis.

4) Can the authors also report the HR factor needed to obtain agreement with the monomer spectrum (bottom of page 7).

We have added in Page 8 the HR factor (0.48) for the bright excitation of the isolated monomer.

5) Some simple spelling: pg 4 last paragraph "tunned" also "Becke Jonhson"

We thank the reviewer for pointing out the spelling errors in Page 4. We have corrected them.
